# Vibration and Buckling of Shear Deformable Functionally Graded Nanoporous Metal Foam Nanoshells

**DOI:** 10.3390/nano9020271

**Published:** 2019-02-15

**Authors:** Yufei Zhang, Fei Zhang

**Affiliations:** 1 College of Aerospace Engineering, Shenyang Aerospace University, Shenyang 110136, China; 2 College of Sciences, Northeastern University, Shenyang 110819, China

**Keywords:** nanoporous metal foam, nanoshell, buckling, free vibration, strain gradient theory, first-order shear deformation theory

## Abstract

This article aims to investigate free vibration and buckling of functionally graded (FG) nanoporous metal foam (NPMF) nanoshells. The first-order shear deformation (FSD) shell theory is adopted and the theoretical model is formulated by using Mindlin’s most general strain gradient theory, which can derive several well-known simplified models. The symmetric and unsymmetric nanoporosity distributions are considered for the structural composition. Hamilton’s principle is employed to deduce the governing equations as well as the boundary conditions. Then, via the Navier solution technique, an analytical solution for the free vibration and buckling of FG NPMF nanoshells is presented. Afterwards, a detailed parametric analysis is conducted to highlight the effects of the nanoporosity coefficient, nanoporosity distribution, length scale parameter, and geometrical parameters on the mechanical behaviors of FG NPMF nanoshells.

## 1. Introduction

Functionally graded materials (FGMs) have a continuous and smooth graded distribution of material properties in the spatial field. Due to their superior properties and advantages, FGMs have been successfully extended to various engineering applications and received much attention [1,2,3,4,5,6,7,8,9,10,11,12,13,14,15,16,17,18,19,20,21,22,23,24]. Recently, a breakthrough made it possible to realize desired structural properties by adjusting the local density of structures, thereby developing novel functionally graded (FG) porous structures composed of metal foams having graded density [25,26,27,28]. The application of nanoporous metal foams (NPMFs) has been extended to some advanced engineering fields due to their extremely high specific surface area [29,30,31,32]. This kind of material has a combination of properties that is not achievable for ceramics, metals, or dense polymers.

Micro/nanostructures have been successfully used in shape memory alloys [33] and micro- and nano-electro-mechanical systems (MEMS and NEMS) [34,35]. The small-scale effects on the mechanical behaviors of micro/nanostructures have been experimentally observed in their applications [36,37]. It was revealed that the mechanical behaviors of micro/nanostructures were different from their macro counterparts due to the size effect [38,39]. Due to the lack of intrinsic material length scale parameters, the classical continuum theory has no ability to predict the mechanical characteristics of micro/nanostructures. Therefore, several size-dependent continuum theories have been proposed to compensate for the drawbacks of the classical continuum theory for micro/nanostructures. One of the size-dependent continuum theories is Mindlin’s strain gradient theory (SGT) [40], which is known as the general form of the SGT containing five additional material length scale parameters compared to the classical continuum theory. Later, several special forms of Mindlin’s SGT were proposed. For instance, one of the most popular forms is the modified strain gradient theory (MSGT) [37]. In fact, this theory is a more useful form of Mindlin’s SGT including three material length scale parameters related to symmetric rotation gradients, deviatoric stretch gradients, and dilatation gradients. Several successful applications of the MSGT in dynamic and static analyses of micro/nanobeams [41,42,43,44], plates [45,46,47], and shells [48,49,50] have been reported. It should be noticed that the modified couple stress theory (MCST) [51] can be achieved by ignoring two of the three material length scale parameters in the MSGT. Moreover, the MSGT can be simplified to the classical theory (CT) by neglecting all of the three material length scale parameters.

Recently, the structural performance of NPMF micro/nanobeams has been investigated by several researchers. Post-buckling analysis for nanobeams made of NPMFs is presented by Barati and Zenkour [52] via the nonlocal elasticity theory (NET). By using the nonlocal strain gradient theory together with the third-order shear deformation beam theory, nonlinear bending of FG NPMF micro/nanobeams reinforced by graphene platelets has been analyzed by Sahmani et al. [53]. Wang et al. [54] utilized the sinusoidal beam theory and the MSGT to study the vibration and bending of NPMF microbeams.

Shell-type structures have excellent mechanical properties [55,56,57,58,59,60,61], and thus, nanoshells are important components in various MEMS and NEMS [62,63,64]. Complete knowledge of the mechanical properties of nanoshells encourages researchers to use them more efficiently. Therefore, some research has been made to illustrate the buckling and vibration characteristics of nanoshells. For example, by using the NET, Hoseinzadeh and Khadem [65] investigated the thermoelastic vibration of double-walled carbon nanotubes (CNTs). By employing the classical shell theory together with the Gurtin-Murdoch elasticity theory, Sahmani et al. [66] analyzed the postbuckling and nonlinear buckling of cylindrical nanoshells subjected to radial and axial compressive loads. Implementing the NET in the first-order shear deformation (FSD) shell theory, Ansari et al. [67] explored the buckling behavior of multi-walled CNTs including the effect of the thermal environment. Wang et al. [68] studied the nonlinear vibration of nanoshells conveying fluid based on the surface stress elasticity theory as well as the classical shell theory.

In the present study, we aim to make an attempt to investigate the vibration and buckling of circular cylindrical nanoshells made from FG NPMFs. In order to accommodate the size dependency of the nanostructure, the general SGT is used to develop the size-dependent first-order shear deformable nanoporous nanoshell model. The governing equations, as well as the related boundary conditions, are obtained simultaneously by utilizing Hamilton’s principle. The free vibration and axial buckling of simply supported nanoporous circular cylindrical nanoshells are solved analytically by means of the Navier solution technique. Moreover, the influence of some key parameters on the vibration and buckling properties of the system is shown.

## 2. FG NPMF Circular Cylindrical Nanoshells

**Figure 1:**
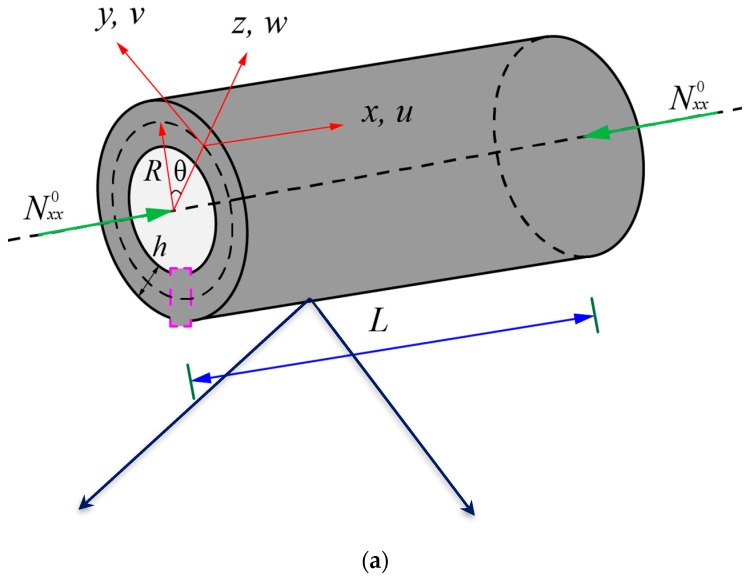

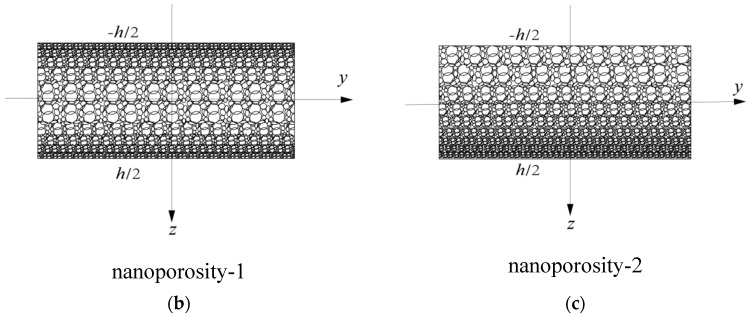
Schematic of a functionally graded (FG) nanoporous metal foam (NPMF) cylindrical nanoshell. (**a**) Coordinate system; (**b**) nanoporosity-1; (**c**) nanoporosity-2.

An FG NPMF circular cylindrical nanoshell of middle-surface radius *R*, thickness *h*, and length *L* is shown in Figure 1. Two kinds of nanoporosity distribution in the thickness direction are considered, namely, nanoporosity-1 and nanoporosity-2. Additionally, the nanoshell is subjected to axial loads $N_{xx}^{0}$.

Owing to non-uniform nanoporosity distribution, mass densities *ρ*(*z*), Young’s modulus *E*(*z*), and shear modulus *μ*(*z*) of the nanoshell are functions of position and can be written as [69,70,71,72,73,74]:

Nanoporosity-1:

(1) $$E(z)=E_{1}^{*}\left[1-e_{0}\cos\left(\pi \zeta \right)\right]$$

(2) $$\rho (z)=\rho_{1}^{*}\left[1-e_{m}\cos\left(\pi \zeta \right)\right]$$

(3) $$\mu (z)=\mu_{1}^{*}\left[1-e_{0}\cos\left(\pi \zeta \right)\right]$$

Nanoporosity-2:

(4) $$E(z)=E_{1}^{*}\left[1-e_{0}\cos\left(\frac{\pi \zeta }{2}+\frac{\pi }{4}\right)\right]$$

(5) $$\rho (z)=\rho_{1}^{*}\left[1-e_{m}\cos\left(\frac{\pi \zeta }{2}+\frac{\pi }{4}\right)\right]$$

(6) $$\mu (z)=\mu_{1}^{*}\left[1-e_{0}\cos\left(\frac{\pi \zeta }{2}+\frac{\pi }{4}\right)\right]$$

where *ζ* = *z*/*h*, the nanoporosity coefficients are $e_{0}{=1-E}_{0}^{*}{/E}_{1}^{*}\;(0\;\leq {\;e}_{0}\;<1)$ and $e_{m}=1-\rho_{0}^{*}{/\rho }_{1}^{*}\;(0\;\leq {\;e}_{m}\;<\;1)$, $\rho_{0}^{*}$ and $\rho_{1}^{*}$ are the minimum and maximum values of the mass density, respectively. The minimum Young’s modulus $E_{0}^{*}$ and the maximum value $E_{1}^{*}$ are related to the minimum shear modulus $\mu_{0}^{*}$ and the maximum value $\mu_{1}^{*}$ according to $\mu_{i}^{*}{=E}_{i}^{*}/[2(1+\nu )]\;(i=0,\;1)$, in which *ν* indicates Poisson’s ratio.

For an open-cell metal foam, we have [75,76]:

(7) $$\frac{E_{0}^{*}}{E_{1}^{*}}={\left(\frac{\rho_{0}^{*}}{\rho_{1}^{*}}\right)}^{2}$$

Thus, the relation between $e_{0}$ and $e_{m}$ is obtained as:

(8) $$e_{m}=1-\sqrt{1-e_{0}}$$

**Figure 2:**
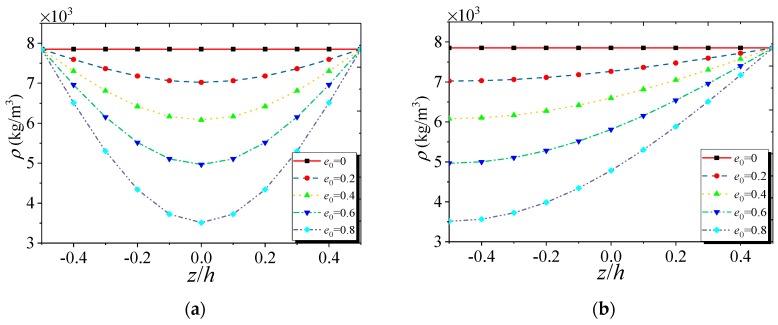
Variation of mass density of FG NPMF nanoshell: (**a**) nanoporosity-1; (**b**) nanoporosity-2.

**Figure 3:**
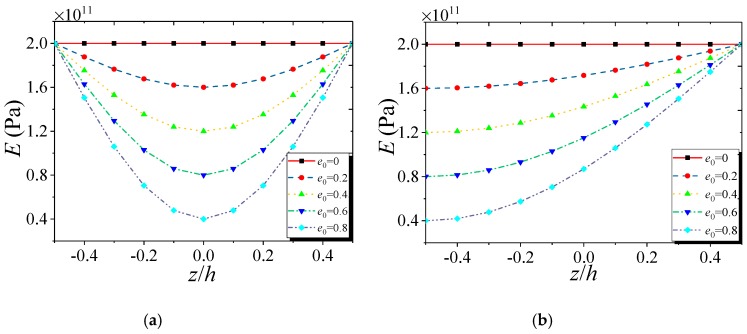
Variation of Young’s modulus of FG NPMF nanoshell: (**a**) nanoporosity-1; (**b**) nanoporosity-2.

Figure 2 and Figure 3 give the variations of mass density and Young’s modulus, respectively, of the FG NPMF nanoshell along the thickness direction. Note that both kinds of nanoporosity distribution have the same minimum and maximum values of mass density and elasticity modulus. For the nanoporosity-1 nanoshell, it possesses the minimum values of mass density and Young’s modulus on the middle surface (*z* = 0) of the nanoshell; while the maximum values are on the outer (*z* = *h*/2) and inner (*z* = −*h*/2) surfaces which are equal to the values of the nanoshell that consisted of solid metal. For the nanoporosity-2, mass density and elasticity modulus have the minimum values on the inner surface and gradually increase to the maximum values on the outer surface of the shell.

## 3. Theory and Formulation

### 3.1. General SGT

As we know, the strain energy density in the CT is described as the function of the strain tensor **ε**. The strain energy density in Mindlin’s SGT, however, also incorporates the third-order strain gradient tensor **ξ**. Therefore, the strain energy density *W* has the most general form [40,77]:

(9) $$W(\varepsilon ,\xi )=\frac{1}{2}\lambda \varepsilon_{ii}\varepsilon_{jj}+\mu \varepsilon_{ij}\varepsilon_{ij}+a_{1}\xi_{ikk}\xi_{ijj}+a_{2}\xi_{kjj}\xi_{iik}+a_{3}\xi_{jjk}\xi_{iik}+a_{4}\xi_{ijk}\xi_{ijk}+a_{5}\xi_{kji}\xi_{ijk}$$

in which *a_i_* (*i* = 1, 2, …, 5) are additional constants which can accommodate the small-scale effect of micro/nanostructures, and *λ* is Lame’s first parameter defined as [78,79]:

(10) $$\lambda =\frac{E\nu }{\left(1+\nu )(1-2\nu \right)}$$

In Equation (9), the third-order strain gradient tensor **ξ** and infinitesimal strain tensor **ε** are defined as [40]:

(11) $$\begin{array}{cc} \varepsilon =\frac{1}{2}\left(\nabla u+{\left(\nabla u\right)}^{T}\right), & \varepsilon_{ij}=\varepsilon_{ji}=\frac{1}{2}\left(u_{i,j}+u_{j,i}\right) \end{array}$$

(12) $${\begin{array}{cc} \xi =\nabla \varepsilon ,\; & \xi_{ijk}=\xi_{ikj}=\varepsilon_{jk,i}=\frac{1}{2}\left(u_{j,k}+u_{k,j}\right) \end{array}}_{,i}$$

in which **u** represents the displacement vector and ∇ is gradient operator.

The double stress tensor *τ_ijk_* and Cauchy stress tensor *σ_ij_* are written as [80]:

(13) $$\sigma_{ij}=\sigma_{ji}=\frac{\partial W}{\partial \varepsilon_{ij}}=\lambda \;\varepsilon_{kk}\;\delta_{ij}+2\mu \;\varepsilon_{ij}$$

(14) $$\begin{array}{l} \tau_{ijk}=\tau_{ikj}=\frac{\partial W}{\partial \xi_{ijk}}=\frac{a_{1}}{2}\left(\xi_{jpp}\delta_{ik}+2\xi_{ppi}\delta_{jk}+\xi_{kpp}\delta_{ij}\right)+2a_{2}\xi_{ipp}\delta_{jk}+a_{3}\left(\xi_{ppj}\delta_{ik}+\xi_{ppk}\delta_{ij}\right)\\ +2a_{4}\xi_{ijk}+a_{5}\left(\xi_{kji}+\xi_{jki}\right) \end{array}$$

where *δ_ij_* represent the Kronecker delta.

### 3.2. Constitutive Relations and Strain Energy

The displacement field for the FG NPMF cylindrical nanoshell according to the FSD shell theory can be defined as [81,82,83,84,85]:

(15) $$\left\{\begin{array}{l} u_{x}\left(x,y,z,t\right)\\ u_{y}\left(x,y,z,t\right)\\ u_{z}\left(x,y,z,t\right) \end{array}\right\}=\left\{\begin{array}{l} u(x,y,t)\\ v(x,y,t)\\ w(x,y,t) \end{array}\right\}+z\left\{\begin{array}{c} \psi_{x}(x,y,t)\\ \psi_{y}(x,y,t)\\ 0 \end{array}\right\}$$

In Equation (15), *u_x_*, *u_y_*, and *u_z_* stand for the displacements of any point in the nanoshell along the *x*, *y*, and *z* directions, respectively; *u*, *v*, and *w* denote displacement components of a point at the middle surface; *ψ_x_* and *ψ_y_* are the rotations of the transverse normals about the *y* and *x* axes, respectively; and *t* denotes time.

The nonzero constituents of strain tensor **ε** are given by: [86,87]

(16) $$\left\{ \begin{array}{l} \varepsilon_{xx}=\phi_{0}+z\phi_{1},\\ \varepsilon_{yy}=\varphi_{0}+z\varphi_{1},\\ \varepsilon_{xy}=\varepsilon_{yx}=\left(k_{0}+zk_{1}\right)/2,\\ \varepsilon_{yz}=\varepsilon_{zy}=\gamma_{2}/2,\\ \varepsilon_{xz}=\varepsilon_{zx}=\gamma_{1}/2. \end{array}\right.$$

where *ϕ*_0_, *φ*_0_, and *k*_0_ are the middle surface strains, *ϕ*_1_, *φ*_1_, and *k*_1_ are changes in the curvature and torsion of the middle surface, and *γ*_1_ and *γ*_2_ are the transverse shear strains. They are given by:

(17) $$\left\{ \begin{array}{l} \phi_{0}=\frac{\partial u}{\partial x},\phi_{1}=\frac{\partial \psi_{x}}{\partial x},\varphi_{0}=\frac{w}{R}+\frac{\partial v}{\partial y},\varphi_{1}=\frac{\partial \psi_{y}}{\partial y},k_{0}=\frac{\partial v}{\partial x}+\frac{\partial u}{\partial y},\\ k_{1}=\frac{\partial \psi_{y}}{\partial x}+\frac{\partial \psi_{x}}{\partial y},\gamma_{1}=\frac{\partial w}{\partial x}+\psi_{x},\gamma_{2}=\psi_{y}-\frac{v}{R}+\frac{\partial w}{\partial y}. \end{array}\right.$$

According to Equation (12), the following nonzero constituents of strain gradient tensor **ξ** are obtained:

(18) $$\left\{ \begin{array}{l} \xi_{xxx}=\frac{\partial \phi_{0}}{\partial x}+z\frac{\partial \phi_{1}}{\partial x},\xi_{yyy}=\frac{\partial \varphi_{0}}{\partial y}+z\frac{\partial \varphi_{1}}{\partial y},\xi_{xyy}=\frac{\partial \varphi_{0}}{\partial x}+z\frac{\partial \varphi_{1}}{\partial x},\\ \xi_{zyy}=\varphi_{1},\xi_{yxx}=\frac{\partial \phi_{0}}{\partial y}+z\frac{\partial \phi_{1}}{\partial y},\xi_{xxy}=\xi_{xyx}=\frac{1}{2}\left(\frac{\partial k_{0}}{\partial x}+z\frac{\partial k_{1}}{\partial x}\right),\xi_{zxx}=\phi_{1},\\ \xi_{yxy}=\xi_{yyx}=\frac{1}{2}\left(\frac{\partial k_{0}}{\partial y}+z\frac{\partial k_{1}}{\partial y}\right),\xi_{zxy}=\xi_{zyx}=\frac{k_{1}}{2},\xi_{xxz}=\xi_{xzx}=\frac{1}{2}\frac{\partial \gamma_{1}}{\partial x},\\ \xi_{yxz}=\xi_{yzx}=\frac{1}{2}\frac{\partial \gamma_{1}}{\partial y},\xi_{xyz}=\xi_{xzy}=\frac{1}{2}\frac{\partial \gamma_{2}}{\partial x},\xi_{yyz}=\xi_{yzy}=\frac{1}{2}\frac{\partial \gamma_{2}}{\partial y}. \end{array}\right.$$

By inserting Equations (16) and (18) into Equations (13) and (14), one can get the nonzero constituents of **σ** and **τ** as follows:

(19) $$\left\{ \begin{array}{l} \sigma_{xx}=\left(\lambda +2\mu \right)\left(\phi_{0}+z\phi_{1}\right)+\lambda \left(\varphi_{0}+z\varphi_{1}\right),\\ \sigma_{yy}=\left(\lambda +2\mu \right)\left(\varphi_{0}+z\varphi_{1}\right)+\lambda \left(\phi_{0}+z\phi_{1}\right),\\ \sigma_{xy}=\sigma_{yx}=\mu \left(k_{0}+zk_{1}\right),\\ \sigma_{xz}=\sigma_{zx}=\mu \gamma_{1},\\ \sigma_{yz}=\sigma_{zy}=\mu \gamma_{2}. \end{array}\right.$$

(20) $$\left\{ \begin{array}{l} \tau_{xxx}=\beta_{1}\xi_{xxx}+\beta_{2}\xi_{xyy}+\beta_{3}\xi_{yxy},\\ \tau_{yxx}=\beta_{2}\xi_{yyy}+\beta_{5}\xi_{yxx}+\beta_{4}\xi_{xxy},\\ \tau_{zxx}=\beta_{5}\xi_{zxx}+\beta_{4}\xi_{xxz}+a_{1}\xi_{yyz}+2a_{2}\xi_{zyy},\\ \tau_{xyy}=\beta_{4}\xi_{yxy}+\beta_{2}\xi_{xxx}+\beta_{5}\xi_{xyy},\\ \tau_{yyy}=\beta_{2}\xi_{yxx}+\beta_{3}\xi_{xxy}+\beta_{1}\xi_{yyy},\\ \tau_{zyy}=a_{1}\xi_{xxz}+2a_{2}\xi_{zxx}+\beta_{5}\xi_{zyy}+\beta_{4}\xi_{yyz},\\ \tau_{yzz}=a_{1}\xi_{xxy}+2a_{2}\xi_{yxx}+\beta_{2}\xi_{yyy},\\ \tau_{xxy}=\tau_{xyx}=\frac{\beta_{3}}{2}\xi_{yyy}+\beta_{6}\xi_{xxy}+\frac{\beta_{4}}{2}\xi_{yxx},\\ \tau_{yxy}=\tau_{yyx}=\frac{\beta_{4}}{2}\xi_{xyy}+\frac{\beta_{3}}{2}\xi_{xxx}+\beta_{6}\xi_{yxy},\\ \tau_{zxy}=\tau_{zyx}=2a_{4}\xi_{zxy}+a_{5}\left(\xi_{xyz}+\xi_{yxz}\right),\\ \tau_{xxz}=\tau_{xzx}=\frac{a_{1}}{2}\xi_{zyy}+a_{3}\xi_{yyz}+\beta_{6}\xi_{xxz}+\frac{\beta_{4}}{2}\xi_{zxx},\\ \tau_{yxz}=\tau_{yzx}=2a_{4}\xi_{yxz}+a_{5}\left(\xi_{xyz}+\xi_{zxy}\right),\\ \tau_{xyz}=\tau_{xzy}=2a_{4}\xi_{xyz}+a_{5}\left(\xi_{yxz}+\xi_{zxy}\right),\\ \tau_{yyz}=\tau_{yzy}=\frac{a_{1}}{2}\xi_{zxx}+a_{3}\xi_{xxz}+\beta_{6}\xi_{yyz}+\frac{\beta_{4}}{2}\xi_{zyy}. \end{array}\right.$$

in which

(21) $$\left\{ \begin{array}{ll} \beta_{1}=2\left(a_{1}+a_{2}+a_{3}+a_{4}+a_{5}\right), & \beta_{2}=a_{1}+2a_{2},\\ \beta_{3}=a_{1}+2a_{3}, & \beta_{4}=a_{1}+2a_{5},\\ \beta_{5}=2\left(a_{2}+a_{4}\right), & \beta_{6}=a_{3}+2a_{4}+a_{5}. \end{array}\right.$$

Based on the general SGT, the stored strain energy, $\Pi_{S}$, in a linear elastic material occupying volume *V* can be given by [77]:

(22) $$\Pi_{S}=\frac{1}{2}\int_{V}\left(\sigma_{ij}\varepsilon_{ij}+\tau_{ijk}\xi_{ijk}\right)dV$$

If the strain energy is symbolized by classical part $\Pi_{C}$ and non-classical part $\Pi_{NC}$, the total strain energy is expressed as:

(23) $$\Pi_{S}=\Pi_{NC}+\Pi_{C}$$

in which,

(24) $$\Pi_{C}=\frac{1}{2}\int_{A}\left(N_{xx}\phi_{0}+M_{xx}\phi_{1}+N_{xy}k_{0}+M_{xy}k_{1}+N_{yy}\varphi_{0}+M_{yy}\varphi_{1}+Q_{x}\gamma_{1}+Q_{y}\gamma_{2}\right)dA$$

(25) $$\begin{array}{ll} \Pi_{NC}= & \frac{1}{2}\int_{A}(T_{xxx}\frac{\partial \phi_{0}}{\partial x}+M_{xxx}\frac{\partial \phi_{1}}{\partial x}+T_{yxx}\frac{\partial \phi_{0}}{\partial y}+M_{yxx}\frac{\partial \phi_{1}}{\partial y}+T_{zxx}\phi_{1}\\ & +T_{xyy}\frac{\partial \varphi_{0}}{\partial x}+M_{xyy}\frac{\partial \varphi_{1}}{\partial x}+T_{yyy}\frac{\partial \varphi_{0}}{\partial y}+M_{yyy}\frac{\partial \varphi_{1}}{\partial y}\\ & +T_{zyy}\varphi_{1}+T_{xxy}\frac{\partial k_{0}}{\partial x}+M_{xxy}\frac{\partial k_{1}}{\partial x}+T_{yyx}\frac{\partial k_{0}}{\partial y}\\ & +M_{yyx}\frac{\partial k_{1}}{\partial y}+T_{zxy}k_{1}+T_{xxz}\frac{\partial \gamma_{1}}{\partial x}+T_{yxz}\frac{\partial \gamma_{1}}{\partial y}\\ & +T_{xyz}\frac{\partial \gamma_{2}}{\partial x}+T_{yyz}\frac{\partial \gamma_{2}}{\partial y})dA \end{array}$$

In Equations (24) and (25), the non-classical and classical resultant moments and forces are expressed as follows:

(26) $$\begin{array}{l} N_{ij}=\int_{-\frac{h}{2}}^{\frac{h}{2}}\sigma_{ij}dz,\;\;\;\;\;\;M_{ij}=\int_{-\frac{h}{2}}^{\frac{h}{2}}\sigma_{ij}zdz,\;\;\;\;\;\;Q_{i}=K_{S}\int_{-\frac{h}{2}}^{\frac{h}{2}}\sigma_{iz}dz,\\ T_{ijk}=\int_{-\frac{h}{2}}^{\frac{h}{2}}\tau_{ijk}dz,\;\;\;\;\;\;M_{ijk}=\int_{-\frac{h}{2}}^{\frac{h}{2}}\tau_{ijk}zdz. \end{array}$$

where *K_S_* = 5/6 denotes the shear correction factor [88,89,90,91]; the non-classical and classical resultant moments and forces are given in Appendix A in detail.

### 3.3. Kinetic Energy and External Work

According to the FSD shell theory, the kinetic energy of the FG NPMF nanoshell, $\Pi_{T}$, is written as:

(27) $$\begin{array}{l} \Pi_{T}=\frac{1}{2}\int_{A}\int_{-\frac{h}{2}}^{\frac{h}{2}}\rho (z)\left[{\left(\frac{\partial u}{\partial t}+z\frac{\partial \psi_{x}}{\partial t}\right)}^{2}+{\left(\frac{\partial v}{\partial t}+z\frac{\partial \psi_{y}}{\partial t}\right)}^{2}+{\left(\frac{\partial w}{\partial t}\right)}^{2}\right]dzdA\\ \;\;\;\;=\frac{1}{2}\int_{A}[I_{0}{\left(\frac{\partial u}{\partial t}\right)}^{2}+2I_{1}\left(\frac{\partial u}{\partial t}\right)\left(\frac{\partial \psi_{x}}{\partial t}\right)+I_{0}{\left(\frac{\partial v}{\partial t}\right)}^{2}+I_{0}{\left(\frac{\partial w}{\partial t}\right)}^{2}\\ \;\;\;\;\;\;\;\;\;\;+2I_{1}\left(\frac{\partial v}{\partial t}\right)\left(\frac{\partial \psi_{y}}{\partial t}\right)+I_{2}{\left(\frac{\partial \psi_{x}}{\partial t}\right)}^{2}+I_{2}{\left(\frac{\partial \psi_{y}}{\partial t}\right)}^{2}]dA \end{array}$$

in which

(28) $$\begin{array}{cc} I_{0}=\int_{-\frac{h}{2}}^{\frac{h}{2}}\rho (z)dz, & I_{1}=\int_{-\frac{h}{2}}^{\frac{h}{2}}\rho (z)zdz,I_{2} \end{array}=\int_{-\frac{h}{2}}^{\frac{h}{2}}\rho (z)z^{2}dz.$$

Furthermore, the work $\Pi_{P}$ carried out by axial loads $N_{xx}^{0}$ can be written as:

(29) $$\Pi_{p}=\frac{1}{2}\int_{A}\left[N_{xx}^{0}{\left(\frac{\partial w}{\partial x}\right)}^{2}\right]dA$$

### 3.4. Variational Formulation

Using Hamilton’s principle,

(30) $$\delta \int_{0}^{t}\left(\Pi_{T}-\Pi_{S}-\Pi_{P}\right)dt=0$$

Inserting Equations (23), (27) and (29) into Equation (30) yields the following governing equations:

(31) $$\delta u:\frac{\partial {\bar{N}}_{xx}}{\partial x}+\frac{\partial {\bar{N}}_{xy}}{\partial y}=I_{0}\frac{\partial^{2}u}{\partial t^{2}}+I_{1}\frac{\partial^{2}\psi_{x}}{\partial t^{2}}$$

(32) $$\delta v:\frac{\partial {\bar{N}}_{xy}}{\partial x}+\frac{\partial {\bar{N}}_{yy}}{\partial y}+\frac{{\bar{Q}}_{y}}{R}=I_{0}\frac{\partial^{2}v}{\partial t^{2}}+I_{1}\frac{\partial^{2}\psi_{y}}{\partial t^{2}}$$

(33) $$\delta w:\frac{\partial {\bar{Q}}_{x}}{\partial x}+\frac{\partial {\bar{Q}}_{y}}{\partial y}-\frac{{\bar{N}}_{yy}}{R}+N_{xx}^{0}\frac{\partial^{2}w}{\partial x^{2}}=I_{0}\frac{\partial^{2}w}{\partial t^{2}}$$

(34) $$\delta \psi_{x}:\frac{\partial {\bar{M}}_{xx}}{\partial x}+\frac{\partial {\bar{M}}_{xy}}{\partial y}-{\bar{Q}}_{x}=I_{2}\frac{\partial^{2}\psi_{x}}{\partial t^{2}}+I_{1}\frac{\partial^{2}u}{\partial t^{2}}$$

(35) $$\delta \psi_{y}:\frac{\partial {\bar{M}}_{xy}}{\partial x}+\frac{\partial {\bar{M}}_{yy}}{\partial y}-{\bar{Q}}_{y}=I_{2}\frac{\partial^{2}\psi_{y}}{\partial t^{2}}+I_{1}\frac{\partial^{2}v}{\partial t^{2}}$$

where,

(36) $$\left\{ \begin{array}{l} {\bar{N}}_{xx}=N_{xx}-\frac{\partial T_{yxx}}{\partial y}-\frac{\partial T_{xxx}}{\partial x},\\ {\bar{N}}_{xy}=N_{xy}-\frac{\partial T_{yyx}}{\partial y}-\frac{\partial T_{xxy}}{\partial x},\\ {\bar{N}}_{yy}=N_{yy}-\frac{\partial T_{yyy}}{\partial y}-\frac{\partial T_{xyy}}{\partial x}-\frac{T_{yyz}}{R},\\ {\bar{M}}_{xx}=M_{xx}+T_{zxx}-\frac{\partial M_{yxx}}{\partial y}-\frac{\partial M_{xxx}}{\partial x},\\ {\bar{M}}_{yy}=M_{yy}+T_{zyy}-\frac{\partial M_{yyy}}{\partial y}-\frac{\partial M_{xyy}}{\partial x},\\ {\bar{M}}_{xy}=M_{xy}+T_{zxy}-\frac{\partial M_{yyx}}{\partial y}-\frac{\partial M_{xxy}}{\partial x},\\ {\bar{Q}}_{x}=Q_{x}-\frac{\partial T_{yxz}}{\partial y}-\frac{\partial T_{xxz}}{\partial x},\\ {\bar{Q}}_{y}=Q_{y}-\frac{\partial T_{yyz}}{\partial y}-\frac{\partial T_{xyz}}{\partial x}. \end{array}\right.$$

Simultaneously, boundary conditions are derived as:

(37) $$\begin{array}{l} \delta u=0\;\;\mathrm{or}\;\;\left({\bar{N}}_{xx}\right)n_{x}+\left({\bar{N}}_{xy}\right)n_{y}=0,\\ \delta u_{,x}=0\;\;\mathrm{or}\;\;\left(T_{xxx}\right)n_{x}+\left(T_{yxx}\right)n_{y}=0,\\ \delta u_{,y}=0\;\;\mathrm{or}\;\;\left(T_{xxy}\right)n_{x}+\left(T_{yyx}\right)n_{y}=0. \end{array}$$

(38) $$\begin{array}{l} \delta v=0\;\;\mathrm{or}\;\;\left({\bar{N}}_{xy}-\frac{T_{xyz}}{R}\right)n_{x}+\left({\bar{N}}_{yy}-\frac{T_{yyz}}{R}\right)n_{y}=0,\\ \delta v_{,x}=0\;\;\mathrm{or}\;\;\left(T_{xxy}\right)n_{x}+\left(T_{yyx}\right)n_{y}=0,\\ \delta v_{,y}=0\;\;\mathrm{or}\;\;\left(T_{xyy}\right)n_{x}+\left(T_{yyy}\right)n_{y}=0. \end{array}$$

(39) $$\begin{array}{l} \delta w=0\;\;\;\mathrm{or}\;\;\;\left({\bar{Q}}_{x}+\frac{T_{xyy}}{R}\right)n_{x}+\left({\bar{Q}}_{y}+\frac{T_{yyy}}{R}\right)n_{y}=0,\\ \delta w_{,x}=0\;\;\;\mathrm{or}\;\;\;\left(T_{xxz}\right)n_{x}+\left(T_{yxz}\right)n_{y}=0,\\ \delta w_{,y}=0\;\;\;\mathrm{or}\;\;\;\left(T_{xyz}\right)n_{x}+\left(T_{yyz}\right)n_{y}=0. \end{array}$$

(40) $$\begin{array}{l} \delta \psi_{x}=0\;\;\;\mathrm{or}\;\;\;\left({\bar{M}}_{xx}+T_{xxz}\right)n_{x}+\left({\bar{M}}_{xy}+T_{yxz}\right)n_{y}=0,\\ \delta \psi_{x,x}=0\;\;\mathrm{or}\;\;\left(M_{xxx}\right)n_{x}+\left(M_{yxx}\right)n_{y}=0,\\ \delta \psi_{x,y}=0\;\;\mathrm{or}\;\;\left(M_{xxy}\right)n_{x}+\left(M_{yyx}\right)n_{y}=0. \end{array}$$

(41) $$\begin{array}{l} \delta \psi_{y}=0\;\;\mathrm{or}\;\;\left({\bar{M}}_{xy}+T_{xyz}\right)n_{x}+\left({\bar{M}}_{yy}+T_{yyz}\right)n_{y}=0,\\ \delta \psi_{y,x}=0\;\;\mathrm{or}\;\;\left(M_{xxy}\right)n_{x}+\left(M_{yyx}\right)n_{y}=0,\\ \delta \psi_{y,y}=0\;\;\mathrm{or}\;\;\left(M_{xyy}\right)n_{x}+\left(M_{yyy}\right)n_{y}=0. \end{array}$$

where *n_x_* as well as *n_y_* indicate the direction cosines of the outward unit normal to the boundary of the mid-plane.

Substituting Equation (36) into Equations (31)–(35) and considering Equation (17) and Appendix A, it yields the governing equations in terms of *u*, *v*, *w*, *ψ_x_*, and *ψ_y_*:

(42) $$\begin{array}{l} A_{11}\frac{\partial^{2}u}{\partial x^{2}}+A_{55}\left(\frac{\partial^{2}u}{\partial y^{2}}+\frac{\partial^{2}v}{\partial x\partial y}\right)-E_{1}\frac{\partial^{4}u}{\partial x^{4}}-\frac{E_{6}}{2}\frac{\partial^{4}u}{\partial y^{4}}\\ -\left(E_{3}+E_{4}+E_{5}+\frac{E_{6}}{2}\right)\frac{\partial^{4}u}{\partial y^{2}\partial x^{2}}\;-\frac{\left(2E_{2}+E_{3}+E_{4}+E_{6}\right)}{2}\left(\frac{\partial^{4}v}{\partial y\partial x^{3}}+\frac{\partial^{4}v}{\partial y^{3}\partial x}\right)\\ +\left(A_{12}+A_{55}\right)\frac{\partial^{2}v}{\partial y\partial x}+\frac{A_{12}}{R}\frac{\partial w}{\partial x}-\frac{2E_{2}+E_{3}+E_{4}}{2R}\frac{\partial^{3}w}{\partial y^{2}\partial x}\\ -\frac{E_{2}}{R}\frac{\partial^{3}w}{\partial x^{3}}+B_{11}\frac{\partial^{2}\psi_{x}}{\partial x^{2}}+B_{55}\frac{\partial^{2}\psi_{x}}{\partial y^{2}}-\left(F_{3}+F_{4}+F_{5}+\frac{F_{6}}{2}\right)\frac{\partial^{4}\psi_{x}}{\partial y^{2}\partial x^{2}}-F_{1}\frac{\partial^{4}\psi_{x}}{\partial x^{4}}\\ -\frac{F_{6}}{2}\frac{\partial^{4}\psi_{x}}{\partial y^{4}}-\frac{2F_{2}+F_{3}+F_{4}+F_{6}}{2}\left(\frac{\partial^{4}\psi_{y}}{\partial y\partial x^{3}}+\frac{\partial^{4}\psi_{y}}{\partial y^{3}\partial x}\right)\\ +\left(B_{12}+B_{55}\right)\frac{\partial^{2}\psi_{y}}{\partial y\partial x}=I_{0}\frac{\partial^{2}u}{\partial t^{2}}+I_{1}\frac{\partial^{2}\psi_{x}}{\partial t^{2}} \end{array}$$

(43) $$\begin{array}{l} -\frac{2E_{2}+E_{3}+E_{4}+E_{6}}{2}\left(\frac{\partial^{4}u}{\partial y\partial x^{3}}+\frac{\partial^{4}u}{\partial y^{3}\partial x}\right)+\left(A_{12}+A_{55}\right)\frac{\partial^{2}u}{\partial y\partial x}\\ -\left(E_{3}+E_{4}+E_{5}+\frac{E_{6}}{2}\right)\frac{\partial^{4}v}{\partial y^{2}\partial x^{2}}-\frac{E_{6}}{2}\frac{\partial^{4}v}{\partial x^{4}}-E_{1}\frac{\partial^{4}v}{\partial y^{4}}+A_{55}\frac{\partial^{2}v}{\partial x^{2}}\;+A_{11}\frac{\partial^{2}v}{\partial y^{2}}-\frac{K_{S}A_{55}}{R^{2}}v\\ +\frac{1}{R}\left(A_{11}+K_{S}A_{55}\right)\frac{\partial w}{\partial y}-\frac{1}{R}\left(\frac{E_{3}+E_{4}+2E_{5}}{2}+A_{3}+A_{4}+\frac{A_{5}}{2}\right)\frac{\partial^{3}w}{\partial y\partial x^{2}}\\ +\frac{E_{1}+E_{6}}{R}\frac{\partial^{3}w}{\partial y^{3}}-\left[\frac{1}{2R}\left(2A_{1}+2A_{3}+2A_{5}\right)+B_{12}+B_{55}\right]\frac{\partial^{2}\psi_{x}}{\partial y\partial x}\\ -\frac{2F_{2}+F_{3}+F_{4}+F_{6}}{2}\left(\frac{\partial^{4}\psi_{x}}{\partial y^{3}\partial x}+\frac{\partial^{4}\psi_{x}}{\partial y\partial x^{3}}\right)+\left[B_{55}+\frac{1}{2R}\left(2A_{4}+A_{5}\right)\right]\frac{\partial^{2}\psi_{y}}{\partial x^{2}}\\ +\;\left(B_{11}-\frac{E_{4}+E_{6}}{R}\right)\frac{\partial^{2}\psi_{y}}{\partial y^{2}}-F_{1}\frac{\partial^{4}\psi_{y}}{\partial y^{4}}-\frac{F_{6}}{2}\frac{\partial^{4}\psi_{y}}{\partial x^{4}}\\ -\left(F_{3}+F_{4}+F_{5}+\frac{F_{6}}{2}\right)\frac{\partial^{4}\psi_{y}}{\partial y^{2}\partial x^{2}}+\frac{K_{S}A_{55}}{R}\psi_{y}=I_{0}\frac{\partial^{2}v}{\partial t^{2}}+I_{1}\frac{\partial^{2}\psi_{y}}{\partial t^{2}} \end{array}$$

(44) $$\begin{array}{l} -\frac{A_{12}}{R}\frac{\partial u}{\partial x}-\frac{1}{2R}\left(2E_{2}+E_{3}+E_{4}\right)\frac{\partial^{3}u}{\partial y^{2}\partial x}+\frac{E_{2}}{2R}\frac{\partial^{3}u}{\partial x^{3}}\\ -\frac{1}{R}\left(A_{11}+K_{S}A_{55}+\frac{E_{6}}{2R^{2}}\right)\frac{\partial v}{\partial y}+\frac{1}{R}\left(\frac{E_{3}+E_{4}+2E_{5}}{2}+\frac{A_{3}+2A_{4}+A_{5}}{2}\right)\frac{\partial^{3}v}{\partial y\partial x^{2}}\\ +\frac{1}{R}\left(\frac{2E_{1}+E_{6}}{2}\right)\frac{\partial^{3}v}{\partial y^{3}}-\left(A_{3}+2A_{4}+A_{5}\right)\frac{\partial^{4}w}{\partial y^{2}\partial x^{2}}\\ -\frac{E_{6}}{2}\left(\frac{\partial^{4}w}{\partial x^{4}}+\frac{\partial^{4}w}{\partial y^{4}}\right)+\left(A_{55}K_{S}+\frac{2E_{5}+A_{3}}{2R^{2}}\right)\frac{\partial^{2}w}{\partial x^{2}}\\ +\left(A_{55}K_{S}+\frac{2E_{1}+E_{6}}{R^{2}}\right)\frac{\partial^{2}w}{\partial y^{2}}-\frac{A_{11}}{R^{2}}w-\left(\frac{A_{1}-A_{3}-2A_{4}-3A_{5}}{2}\right)\frac{\partial^{3}\psi_{x}}{\partial y^{2}\partial x}\\ -\left(\frac{E_{4}+E_{6}}{2}-\frac{F_{2}}{R}\right)\frac{\partial^{3}\psi_{x}}{\partial x^{3}}+\left(K_{S}A_{55}-\frac{B_{12}}{R}+\frac{A_{1}+A_{3}}{R^{2}}\right)\frac{\partial \psi_{x}}{\partial x}\\ -\left(\frac{A_{1}+A_{3}+2A_{4}+3A_{5}}{2}+\frac{F_{3}+F_{4}+2F_{5}}{R}\right)\frac{\partial^{3}\psi_{y}}{\partial y\partial x^{2}}-\left(\frac{E_{4}+E_{6}}{2}+\frac{-2F_{1}}{R}\right)\frac{\partial^{3}\psi_{y}}{\partial y^{3}}\\ +\left(K_{S}A_{55}-\frac{B_{11}}{R}+\frac{E_{4}+E_{6}}{2R^{2}}\right)\frac{\partial \psi_{y}}{\partial y}+N_{xx}^{0}\frac{\partial^{2}w}{\partial x^{2}}=I_{0}\frac{\partial^{2}w}{\partial t^{2}} \end{array}$$

(45) $$\begin{array}{l} B_{11}\frac{\partial^{2}u}{\partial x^{2}}+B_{55}\frac{\partial^{2}u}{\partial y^{2}}\;-\left(F_{3}+F_{4}+F_{5}+\frac{F_{6}}{2}\right)\frac{\partial^{4}u}{\partial y^{2}\partial x^{2}}\\ -F_{1}\frac{\partial^{4}u}{\partial x^{4}}-\frac{F_{6}}{2}\frac{\partial^{4}u}{\partial y^{4}}+\left[B_{12}+B_{55}-\frac{1}{2R}\left(A_{1}+A_{3}+2A_{5}\right)\right]\frac{\partial^{2}v}{\partial y\partial x}\\ -\frac{2F_{2}+F_{3}+F_{4}+F_{6}}{2}\left(\frac{\partial^{4}v}{\partial y^{3}\partial x}+\frac{\partial^{4}v}{\partial y\partial x^{3}}\right)\;-\left(K_{S}A_{55}-\frac{B_{12}}{R}\right)\frac{\partial w}{\partial x}\\ +\left(\frac{A_{1}+A_{3}+2A_{4}+3A_{5}}{2}\right)\frac{\partial^{3}w}{\partial y^{2}\partial x}+\left(\frac{E_{4}+E_{6}}{2}-\frac{F_{2}}{R}\right)\frac{\partial^{3}w}{\partial x^{3}}\\ -G_{1}\frac{\partial^{4}\psi_{x}}{\partial x^{4}}-\frac{G_{6}}{2}\frac{\partial^{4}\psi_{x}}{\partial y^{4}}-\left(G_{3}+G_{4}+G_{5}+G_{6}\right)\frac{\partial^{4}\psi_{x}}{\partial y^{2}\partial x^{2}}\\ +\left(D_{11}+E_{4}+E_{5}+\frac{E_{6}}{2}\right)\frac{\partial^{2}\psi_{x}}{\partial x^{2}}+\left(D_{55}+2A_{4}+A_{5}\right)\frac{\partial^{2}\psi_{x}}{\partial y^{2}}\\ -A_{55}K_{S}\psi_{x}-\frac{G_{2}+G_{3}+G_{4}+G_{6}}{2}\left(\frac{\partial^{4}\psi_{y}}{\partial y\partial x^{3}}+\frac{\partial^{4}\psi_{y}}{\partial y^{3}\partial x}\right)\\ +\left(D_{12}+D_{55}+A_{1}+2A_{2}+\frac{A_{3}}{2}+A_{4}+\frac{A_{5}}{2}\right)\frac{\partial^{2}\psi_{y}}{\partial y\partial x}=I_{2}\frac{\partial^{2}\psi_{x}}{\partial t^{2}}+I_{1}\frac{\partial^{2}u}{\partial t^{2}} \end{array}$$

(46) $$\begin{array}{l} \left(B_{12}+B_{55}\right)\frac{\partial^{2}u}{\partial y\partial x}-\frac{2F_{2}+F_{3}+F_{4}+F_{6}}{2}\left(\frac{\partial^{4}u}{\partial y\partial x^{3}}+\frac{\partial^{4}u}{\partial y^{3}\partial x}\right)\\ +\left[B_{55}+\frac{1}{2R}\left(-2A_{4}-A_{5}\right)\right]\frac{\partial^{2}v}{\partial x^{2}}+\left(B_{11}-\frac{E_{4}+E_{6}}{2R}\right)\frac{\partial^{2}v}{\partial y^{2}}\\ -F_{1}\frac{\partial^{4}v}{\partial y^{4}}-\frac{F_{6}}{2}\frac{\partial^{4}v}{\partial x^{4}}-\left(F_{3}+F_{4}+F_{5}+\frac{F_{6}}{2}\right)\frac{\partial^{4}v}{\partial y^{2}\partial x^{2}}+\frac{K_{S}A_{55}}{R}v\\ +\left(\frac{A_{1}+A_{3}+2A_{4}-3A_{5}-F_{3}-F_{4}-2F_{5}}{2}\right)\frac{\partial^{3}w}{\partial y\partial x^{2}}+\left(\frac{E_{4}+E_{6}}{2}-\frac{F_{1}}{R}\right)\frac{\partial^{3}w}{\partial y^{3}}\\ -\left(K_{S}A_{55}-\frac{B_{11}}{R}\right)\frac{\partial w}{\partial y}\;-\frac{2G_{2}+G_{3}+G_{4}+G_{6}}{2}\left(\frac{\partial^{4}\psi_{x}}{\partial y\partial x^{3}}+\frac{\partial^{4}\psi_{x}}{\partial y^{3}\partial x}\right)-\frac{G_{6}}{2}\frac{\partial^{4}\psi_{y}}{\partial x^{4}}-G_{1}\frac{\partial^{4}\psi_{y}}{\partial y^{4}}\\ -\left(G_{3}+G_{4}+G_{5}+\frac{G_{6}}{2}\right)\frac{\partial^{4}\psi_{y}}{\partial y^{2}\partial x^{2}}+\left(D_{55}+2A_{4}+A_{5}\right)\frac{\partial^{2}\psi_{y}}{\partial x^{2}}+\left(D_{11}+E_{4}+E_{5}+\frac{E_{6}}{2}\right)\frac{\partial^{2}\psi_{y}}{\partial y^{2}}\;\\ +\left(D_{12}+D_{55}+A_{1}+2A_{2}+\frac{A_{3}}{2}+A_{4}+\frac{3A_{5}}{2}\right)\frac{\partial^{2}\psi_{x}}{\partial y\partial x}-K_{S}A_{55}\psi_{y}=I_{2}\frac{\partial^{2}\psi_{y}}{\partial t^{2}}+I_{1}\frac{\partial^{2}v}{\partial t^{2}} \end{array}$$

in which *A_ij_*, *B_ij_*, *D_ij_*, *A_i_*, *B_i_*, *E_i_*, *F_i_*, and *G_i_* (*i*, *j* = 1, 2, …, 6) are given in Appendix B.

It is worth mentioning that the present general strain gradient nanoshell model can reduce to those of MCST, MSGT, and CT. The MSGT model can be achieved if *a_i_* (*i* = 1, 2, …, 5) are defined by three material length scale parameters as follows:

(47) $$\begin{array}{l} \begin{array}{cc} a_{1}=\mu \left(l_{2}^{2}-\frac{4}{15}l_{1}^{2}\right), & \;\;\;\;\;a_{2}=\mu \left(l_{0}^{2}-\frac{1}{15}l_{1}^{2}-\frac{1}{2}l_{2}^{2}\right), \end{array}\\ \begin{array}{ccc} a_{3}=-\mu \left(\frac{4}{15}l_{1}^{2}+\frac{1}{2}l_{2}^{2}\right), & a_{4}=\mu \left(\frac{1}{3}l_{1}^{2}+l_{2}^{2}\right), & a_{5}=\mu \left(\frac{2}{3}l_{1}^{2}-l_{2}^{2}\right). \end{array} \end{array}$$

where *l*_0_, *l*_1_, and *l*_2_ are material length scale parameters corresponding to dilatation gradients, deviatoric stretch gradients and symmetric rotation gradients, respectively. In the following discussion, we assume that all the material length scale parameters are the same, namely, *l*_0_ = *l*_1_ = *l*_2_ = *l*. In addition, by setting *a*_1_ = *a*_2_ = *a*_3_ = *a*_4_ = *a*_5_ = 0, the present nanoshell model can be simplified to the CT-based model. Moreover, the MCST model [51] can be achieved if *a_i_* (*i* = 1, 2, …, 5) are set as:

(48) $$a_{1}=a_{4}=-2a_{2}=-2a_{3}=-a_{5}=\mu l^{2}$$

## 4. Closed-Form Solution

Herein, we employ the Navier solution technique to analyze the free vibration and axial buckling behaviors of an FG NPMF cylindrical nanoshell. Navier’s method can obtain an analytical solution by introducing the double trigonometric series. Note that this method is only applicable to the simply supported boundary condition. For the other boundary conditions which are different from the simply supported boundary condition, other numerical methods such as the finite element method, differential quadrature method, finite difference method, meshless method, and wavelet method can be used. As an example, the boundary condition of the FG NPMF nanoshell considered in our study is simply supported at edges *x* = 0 as well as *x* = *L*, so one obtains:

(49) $$\left\{ \begin{array}{l} v=w=\psi_{y}={\bar{N}}_{xx}=0,\\ \frac{\partial \psi_{y}}{\partial y}=\frac{\partial \psi_{x}}{\partial x}=\frac{\partial w}{\partial y}=\frac{\partial v}{\partial y}=\frac{\partial u}{\partial x}=0,\\ T_{xxy}=T_{xxz}=M_{xxy}={\bar{M}}_{xx}+T_{xxz}=0. \end{array}\right.$$

The Navier procedure is used by assuming the displacements as follows:

(50) $$\left\{\begin{array}{l} u\left(x,y,t\right)\\ v\left(x,y,t\right)\\ w\left(x,y,t\right)\\ \psi_{x}\left(x,y,t\right)\\ \psi_{y}\left(x,y,t\right) \end{array}\right\}=\sum_{n=1}^{\infty }\sum_{m=1}^{\infty }\left\{\begin{array}{l} u_{mn}\left(t\right)\cos\left(\alpha_{m}x\right)\sin\left(\frac{ny}{R}\right)\\ v_{mn}\left(t\right)\sin\left(\alpha_{m}x\right)\cos\left(\frac{ny}{R}\right)\\ w_{mn}\left(t\right)\sin\left(\alpha_{m}x\right)\sin\left(\frac{ny}{R}\right)\\ \psi_{x}{}_{mn}\left(t\right)\cos\left(\alpha_{m}x\right)\sin\left(\frac{ny}{R}\right)\\ \psi_{y}{}_{mn}\left(t\right)\sin\left(\alpha_{m}x\right)\cos\left(\frac{ny}{R}\right) \end{array}\right\}$$

in which $\alpha_{m}=m\pi /L$, *n* is the circumferential wave number, and *m* is the axial half-wave number. Inserting Equation (50) into Equations (42)–(46) and then eliminating the trigonometric functions, the equations can be re-represented in the matrix form as:

(51) $$M\ddot{d}+\left(K+N_{xx}^{0}K_{g}\right)d=0$$

where the displacement vector **d**, mass matrix **M**, geometric stiffness matrix **K_g_**, and stiffness matrix **K** are:

(52) $$d={\left[u_{mn},v_{mn},w_{mn},\psi_{xmn},\psi_{ymn}\right]}^{T}$$

(53) $$K=\left[\begin{array}{cccc} \begin{array}{c} K_{11}\\ K_{21} \end{array} & \begin{array}{c} K_{12}\\ K_{22} \end{array} & \begin{array}{c} K_{13}\\ K_{23} \end{array} & \begin{array}{c} \begin{array}{cc} K_{14} & K_{15} \end{array}\\ \begin{array}{cc} K_{24} & K_{25} \end{array} \end{array}\\ K_{31} & K_{32} & K_{33} & \begin{array}{cc} K_{34} & K_{35} \end{array}\\ K_{41} & K_{42} & K_{43} & \begin{array}{cc} K_{44} & K_{45} \end{array}\\ K_{51} & K_{52} & K_{53} & \begin{array}{cc} K_{54} & K_{55} \end{array} \end{array}\right]$$

(54) $$M=\left[\begin{array}{cc} \begin{array}{cccc} \begin{array}{c} M_{11}\\ 0 \end{array} & \begin{array}{c} 0\\ M_{22} \end{array} & \begin{array}{c} 0\\ 0 \end{array} & \begin{array}{c} M_{14}\\ 0 \end{array}\\ 0 & 0 & M_{33} & 0\\ M_{41} & 0 & 0 & M_{44}\\ 0 & M_{52} & 0 & 0 \end{array} & \begin{array}{c} \begin{array}{c} 0\\ M_{25} \end{array}\\ 0\\ 0\\ M_{55} \end{array} \end{array}\right]$$

(55) $$K_{g}=\left[\begin{array}{cc} \begin{array}{cccc} \begin{array}{c} 0\\ 0 \end{array} & \begin{array}{c} 0\\ 0 \end{array} & \begin{array}{c} 0\\ 0 \end{array} & \begin{array}{c} 0\\ 0 \end{array}\\ 0 & 0 & K_{g33} & 0\\ 0 & 0 & 0 & 0\\ 0 & 0 & 0 & 0 \end{array} & \begin{array}{c} \begin{array}{c} 0\\ 0 \end{array}\\ 0\\ \begin{array}{c} 0\\ 0 \end{array} \end{array} \end{array}\right]$$

in which the elements in these matrices are given in Appendix C.

If the dynamic displacement is considered, the form of the displacement vector **d** can be written as **d** = d^*^e^i^*^ωt^*. Once we ignore $N_{xx}^{0}$, the eigenvalue problem of free vibrating nanoshells can be obtained as:

(56) $$\left(K-\omega^{2}M\right)d^{*}=0$$

where *ω* represents the natural frequency of the FG NPMF nanoshell. The non-trivial solution requires vanishing of the determinant of the coefficient matrix in Equation (56) [92,93,94,95,96,97,98].

Buckling loads of the FG NPMF nanoshell can be obtained by neglecting the inertia term in Equation (51). Letting $N_{xx}^{0}=-F$, one can get:

(57) $$\left(K-F_{}K_{g}\right)d=0$$

where *F* denotes the buckling load. For different combinations of *m* and *n*, there exists a minimum value which satisfies Equation (57). This minimum value is termed as the critical buckling load *F*_cr_.

## 5. Validation

Some comparative studies are first undertaken to prove the reliability of the present analysis.

### 5.1. Example 1: Homogeneous Cylindrical Nanoshell Based on the MSGT

In Table 1, the present results for a homogeneous simply supported cylindrical nanoshell are compared with those obtained by Zhang et al. [99]. The parameters used are: *E* = 1.06TPa, *ν* = 0.3, *ρ* = 2300 kg/m^3^, *R* = 2.32 nm, and *L*/*R* = 5. The frequency parameter $\bar{\omega }\;=\;\omega R\sqrt{\rho /E}$ of the nanoshell is obtained based on the MSGT. One can see that the results from the current study coincide with those reported in Reference [99].

### 5.2. Example 2: Homogeneous Cylindrical Nanoshells Based on the MCST

In Table 2, the comparison study is conducted for natural frequency $\Omega \;=\;\omega R\sqrt{\rho /E}$ of a homogeneous nanoscale cylindrical shell with a simply supported boundary condition by using the MCST. The adopted material properties are: *E* = 1.06 TPa, *ν* = 0.3, *ρ* = 2300 kg/m^3^. It is observed that the obtained results have a reasonable accordance with those reported [100].

### 5.3. Example 3: FG Cylindrical Shell

Herein, a comparison study is conducted for a simply supported FG cylindrical shell without considering the size effect, as given in Table 3. The FG shell is made of the mixture of Stainless Steel (SS) and Nickel (Ni) with the following material parameters: *E*_SS_ = 207.788 GPa, *ρ*_SS_ = 8166 kg/m^3^ and *ν*_SS_ = 0.317756 for SS, and *E*_Ni_ = 205.098 GPa, *ρ*_Ni_ = 8900 kg/m^3^ and *ν*_ΝI_ = 0.31 for Ni. Our study yields an excellent agreement with Reference [101], bespeaking the correctness of the current research.

## 6. Results and Discussion

In this section, size-dependent free vibration and axial buckling of an FG NPMF nanoshell simply supported at both ends are studied. The material properties of the nanoshell are $E_{1}^{*}\;=\;200\;\mathrm{GPa}$, $\rho_{1}^{*}{\;=\;7850\;\mathrm{kg}/m}^{3}$, and *ν* = 1/3. The dimensionless natural frequency is defined as *Ω* $=\omega R\sqrt{\rho_{1}^{*}/E_{1}^{*}}$ and the dimensionless buckling load is $\bar{F}{=\;F/A}_{110}$, where *A*_110_ is the specific value of *A*_11_ for the homogeneous nanoshell made of solid metal.

### 6.1. Free Vibration Analysis

Table 4 shows the variation of dimensionless natural frequency with the circumferential wave number for various length scale parameters. It is found that by increasing the dimensionless length scale parameter, the natural frequencies of the system decrease. Moreover, the fundamental natural frequency occurs at *n* = 2, independent of the length scale parameter. In the following studies, the mode (1, 2) is chosen as a representative mode.

**Figure 4:**
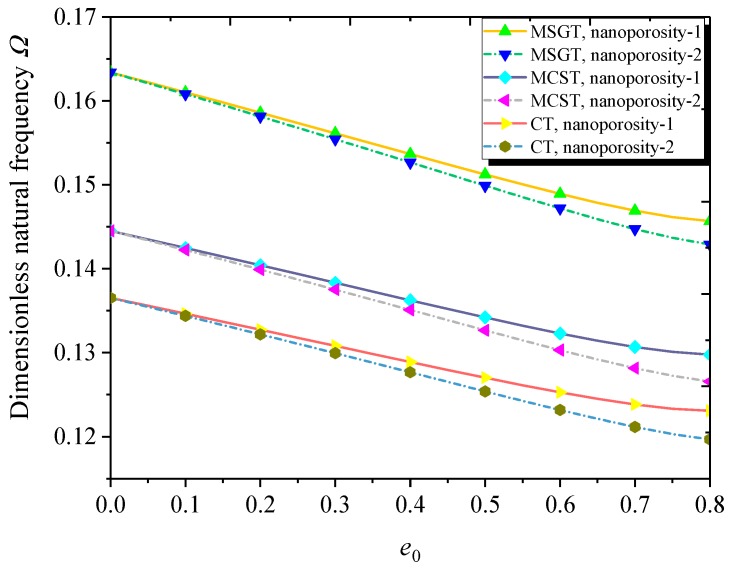
Dimensionless natural frequency versus nanoporosity coefficient with different theories and nanoporosity distributions (*m* = 1, *n* = 2, *h* = 10 nm, *R* = 20 *h*, *h* = 2*l*, *L* = 4*R*).

The dimensionless natural frequency versus nanoporosity coefficient for different theories and nanoporosity distributions is illustrated in Figure 4. Results show that the natural frequency decreases by increasing the nanoporosity coefficient, indicating that the nano-pores decrease the effective stiffness of the nanoshell. Furthermore, the nanoporosity-2 nanoshell has a lower natural frequency than its nanoporosity-1 counterpart. It is observed that the natural frequencies predicted by the MCST and MSGT are greater than the natural frequency predicted by the CT. In other words, the additional length scale parameter makes the FG NPMF nanoshell stiffer. This is due to the extra stiffness introduced in the MCST and MSGT.

**Figure 5:**
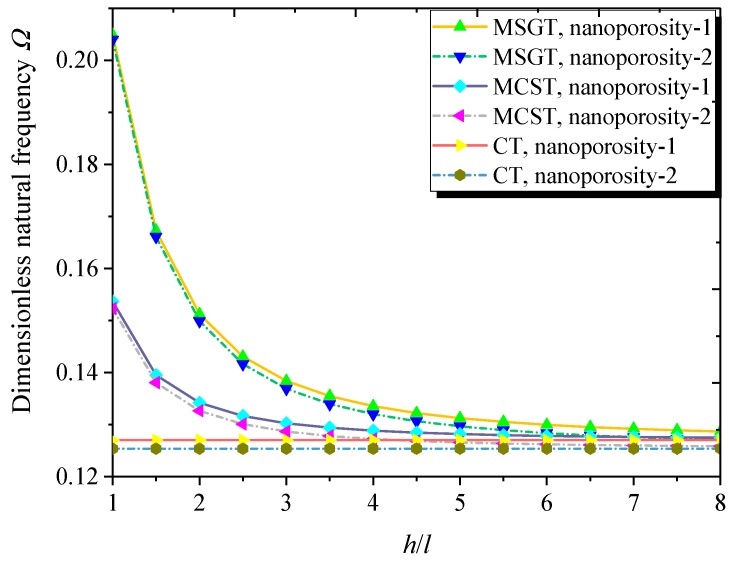
Dimensionless natural frequency versus dimensionless length scale parameter with different theories and nanoporosity distributions (*m* = 1, *n* = 2, *h* = 10 nm, *R* = 20 *h*, *L* = 4*R*, *e*_0_ = 0.5).

Depicted in Figure 5 is the variation of the dimensionless natural frequency against the dimensionless length scale parameter. It is seen that the size effect on natural frequency is more pronounced when the thickness of the nanoshell is comparable to the length scale parameter. The dimensionless natural frequencies from the MCST and MSGT converge to the results from the CT for a large value of the dimensionless length scale parameter, indicating that the larger dimensionless length scale parameter diminishes the size effect on the natural frequency of the FG NPMF nanoshell.

**Figure 6:**
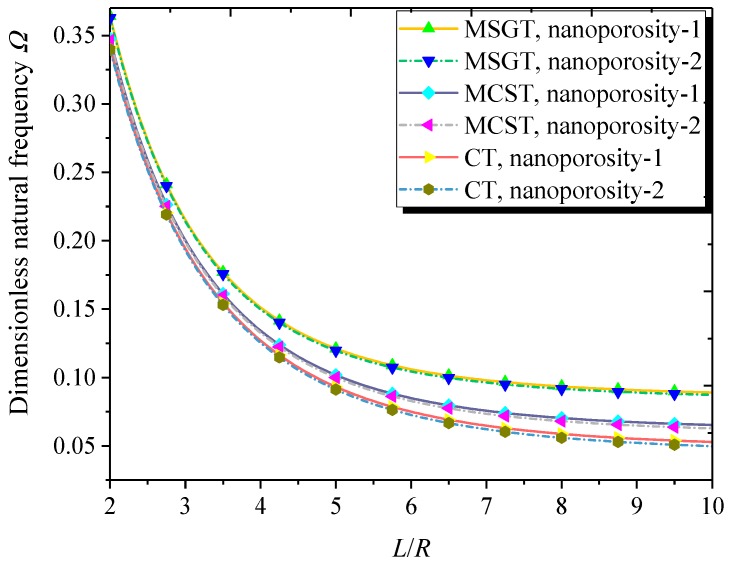
Dimensionless natural frequency versus length-to-radius ratio with different theories and nanoporosity distributions (*m* = 1, *n* = 2, *h* = 10 nm, *R* = 20 *h*, *h* = 2*l*, *e*_0_ = 0.5).

Figure 6 plots the dimensionless natural frequency versus length-to-radius ratio with different theories and nanoporosity distributions. One can see that as the length-to-radius ratio increases, the dimensionless natural frequency decreases gradually. Compared to the MCST, the MSGT leads to more reasonable results due to the introduction of an additional deviatoric stretch gradient tensor and the dilatation gradient tensor in addition to the symmetric rotation gradient tensor.

**Figure 7:**
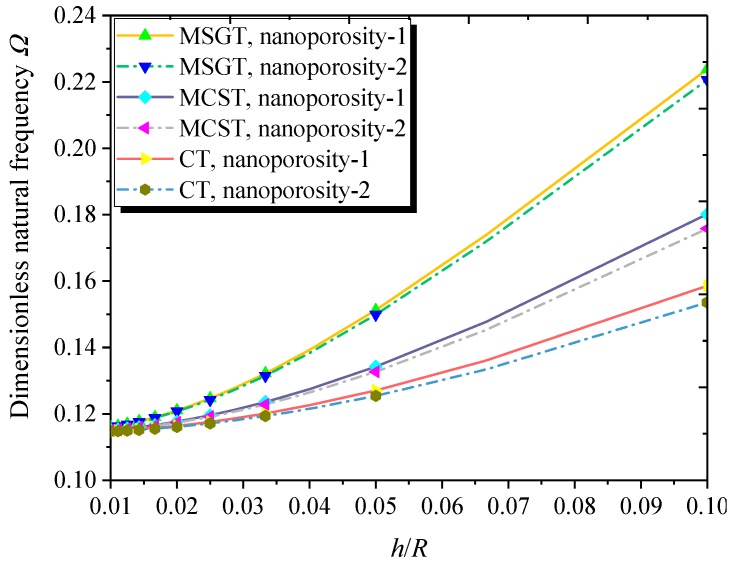
Dimensionless natural frequency versus thickness-to-radius ratio with different theories and nanoporosity distributions (*m* = 1, *n* = 2, *h* = 10 nm, *L* = 4*R*, *h* = 2*l*, *e*_0_ = 0.5).

Figure 7 illustrates the effect of the thickness-to-radius ratio on the dimensionless natural frequency of the FG NPMF nanoshell. As expected, the natural frequency of the FG NPMF nanoshell increases with the rise of thickness-to-radius. This is because the larger thickness-to-radius ratio results in the enhancement of the nanoshell stiffness. Moreover, the difference among the results obtained from the MCST, MSGT, and CT becomes more and more notable as the ratio of thickness-to-radius increases, indicating that the size effect is more significant at the larger thickness-to-radius ratio.

### 6.2. Buckling Analysis

The effect of the length scale parameter on the dimensionless buckling load is shown in Table 5. It is revealed that by increasing the dimensionless length scale parameter, the buckling load of the system decreases. Additionally, with the increase of circumferential wave number, the buckling load first decreases and then increases. It is noted that the critical buckling load occurs at *n* = 2.

**Figure 8:**
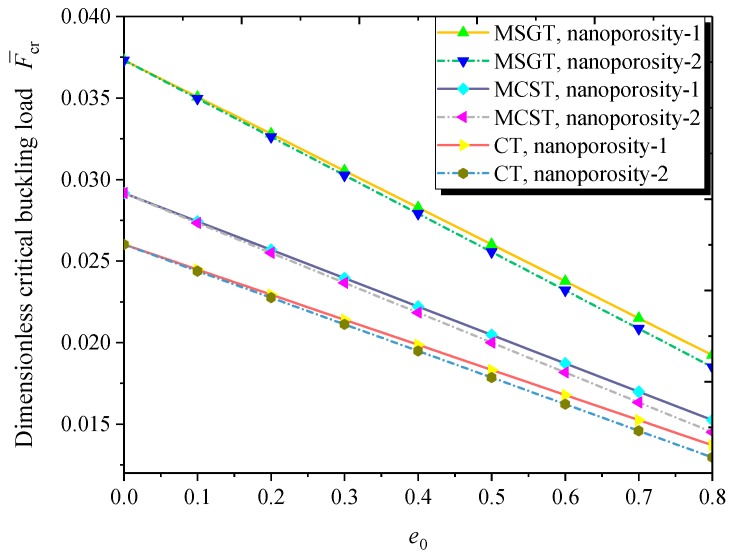
Dimensionless critical buckling load versus nanoporosity coefficient (*m* = 1, *n* = 2, *h* = 10 nm, *R* = 20 *h*, *L* = 4*R*, *h* = 2*l*).

Figure 8 plots the dimensionless critical buckling load versus nanoporosity coefficient for both nanoporosity distributions based on the MCST, MSGT, and CT. As can be observed, the larger nanoporosity coefficient results in a lower dimensionless critical buckling load. Moreover, the nanoporosity-1 nanoshell has a higher critical buckling load than its nanoporosity-2 counterpart. The difference between them tends to be significant with the increase of the nanoporosity coefficient. Furthermore, compared to the MCST, the MSGT leads to a more reasonable buckling load due to the introduction of an additional deviatoric stretch gradient tensor and dilatation gradient tensor in addition to the symmetric rotation gradient tensor.

**Figure 9:**
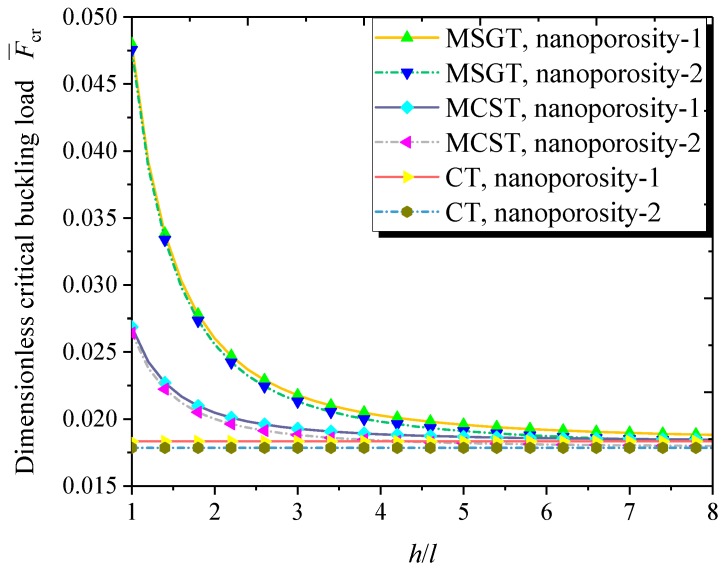
Dimensionless critical buckling load versus dimensionless length scale parameter (*m* = 1, *n* = 2, *h* = 10 nm, *R* = 20 *h*, *L* = 4*R*, *e*_0_ = 0.5).

Figure 9 compares the variation of the dimensionless critical buckling load with the dimensionless length scale parameter based on classical and non-classical shell models. It is noted that the critical buckling load decreases with the increasing dimensionless length scale parameter. In addition, the difference among the results from the three models (MCST, MSGT, and CT) is diminishing when the dimensionless length scale parameter tends to large, indicating that the size effect is only significant when the thickness of the nanoshell is comparable to the length scale parameter. This phenomenon was also found in microplates and microbeams [41,47,102].

**Figure 10:**
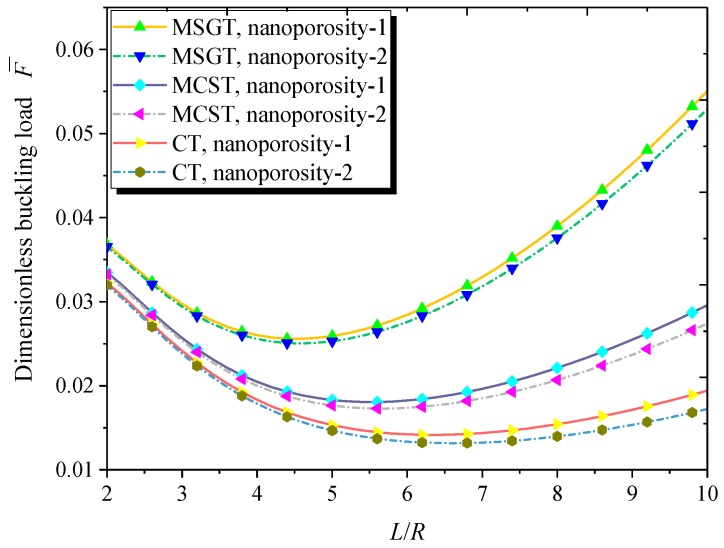
Dimensionless buckling load versus length-to-radius ratio (*m* = 1, *n* = 2, *h* = 10 nm, *R* = 20 *h*, *h* = 2*l*, *e*_0_ = 0.5).

Depicted in Figure 10 is the variation of the dimensionless buckling load with the length-to-radius ratio for both kinds of nanoporosity distribution. It can be seen that with the increase of the length-to-radius ratio, the dimensionless buckling load first decreases and then increases. Moreover, the dimensionless buckling load obtained through the MSGT is greater than those predicted via the CT and MCST. The difference between the results obtained by the MCST, MSGT, and CT becomes more and more significant as the length-to-radius ratio rises.

**Figure 11:**
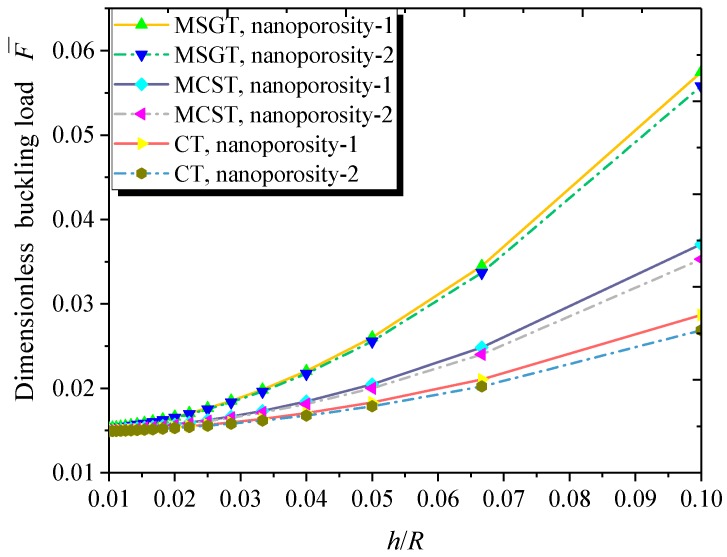
Dimensionless buckling load versus thickness-to-radius ratio (*m* = 1, *n* = 2, *h* = 10 nm, *L* = 4*R*, *h* = 2*l*, *e*_0_ = 0.5).

Figure 11 plots the dimensionless buckling load with respect to the thickness-to-radius ratio for both kinds of nanoporosity distribution. As can be seen, the increase in the thickness-to-radius ratio contributes to the higher buckling load of the FG NPMF nanoshell. This is due to the fact that the larger thickness-to-radius ratio enhances the stiffness of the FG NPMF nanoshell.

## 7. Conclusions

In this paper, size-dependent free vibration and buckling of FG NPMF cylindrical nanoshells are investigated based upon the FSD shell theory and general SGT. The symmetric and unsymmetric nanoporosity distributions are considered for the structural composition. Governing equations, as well as corresponding boundary conditions, are derived via Hamilton’s principle. Moreover, the Navier solution technique is employed to derive the analytical solutions for FG NPMF nanoshells with a simply supported boundary condition. The conclusions can be summarized as follows:

(1) Nanoporosity distribution has a significant influence on the vibration and buckling characteristics of FG NPMF nanoshells. Natural frequencies and buckling loads of the nanoporosity-2 nanoshell are lower than those of the nanoporosity-1 nanoshell. As the nanoporosity coefficient increases, natural frequencies and buckling loads of the nanoshell decrease.
(2) Natural frequencies of the FG NPMF nanoshells decrease with the increasing length-to-radius ratio. Additionally, the larger thickness-to-radius ratio leads to the higher natural frequency of the FG NPMF nanoshell.
(3) Buckling loads decrease first and then increase with the increase of the length-to-radius ratio. Furthermore, buckling loads increase with the increasing thickness-to-radius ratio of the nanoshell.
(4) When the nanoshell thickness is approximately equal to the length scale parameter, the MSGT is more appropriate than the CT and MCST for free vibration and buckling analysis of FG NPMF nanoshells.

## Figures and Tables

**Table 1:** Comparison of dimensionless natural frequency $\bar{\omega }$ for a homogeneous nanoscale cylindrical shell.

(*m*,*n*)	*h*/*R*	*l* = 0			*l* = *h*		
		Zhang et al. [99]	Present	Error (%)	Zhang et al. [99]	Present	Error (%)
(1, 1)	0.02	0.19536	0.19536	0.00	0.19595	0.19561	0.10
	0.05	0.19542	0.19542	0.00	0.19908	0.19694	0.20
	0.1	0.19561	0.19564	0.01	0.20386	0.20148	1.17
(2, 2)	0.02	0.25285	0.25271	0.05	0.27108	0.27004	0.30
	0.05	0.25969	0.25885	0.30	0.35606	0.34641	0.96
	0.1	0.28080	0.27931	0.50	0.50626	0.50145	0.90
(3, 3)	0.02	0.27627	0.27580	0.16	0.37783	0.37382	1.39
	0.05	0.31667	0.31413	0.80	0.71543	0.69918	2.27
	0.1	0.40671	0.41916	2.97	1.08810	1.07892	0.84

**Table 2:** Comparison of dimensionless natural frequency *Ω* for a homogeneous cylindrical nanoshell (*R* = 2.32 nm and *L*/*R* = 5).

*h*/*R*	(*m*, *n*)	*l* = 0			*l* = *h*		
		Ghadiri et al. [100]	Present	Error (%)	Ghadiri et al. [100]	Present	Error (%)
0.02	*m* = *n* = 1	0.19536215	0.19536215	0.00	0.19543206	0.19548050	0.01
	*m* = *n* = 2	0.25271274	0.25271274	0.00	0.25731258	0.25785715	0.09
	*m* = *n* = 3	0.27580092	0.27580092	0.00	0.30621690	0.30717244	0.10
0.05	*m* = *n* = 1	0.19542305	0.19542305	0.00	0.19585782	0.19618570	0.16
	*m* = *n* = 2	0.25884786	0.25884786	0.00	0.28543902	0.28780026	0.80
	*m* = *n* = 3	0.31407326	0.31407326	0.00	0.45457555	0.46000081	1.10

**Table 3:** Comparison of natural frequencies (Hz) for a simply supported FG cylindrical shell (*n* = 1, *R* = 1 m and *L*/*R* = 20).

*h*/*R*	*N*	Loy et al. [101]			Present		
		*m* = 1	*m* = 2	*m* = 3	*m* = 1	*m* = 2	*m* = 3
0.002	0	13.548	4.5920	4.2633	13.548	4.5920	4.2633
	0.5	13.321	4.5168	4.1911	13.321	4.5168	4.1911
	1	13.211	4.4800	4.1569	13.211	4.4800	4.1569
	2	13.103	4.4435	4.1235	13.103	4.4434	4.1234
	5	12.998	4.4068	4.0891	12.998	4.4068	4.0891
0.05	0	13.572	33.296	93.001	13.572	33.242	92.634
	0.5	13.345	32.702	91.319	13.345	32.645	90.943
	1	13.235	32.430	90.553	13.235	32.370	90.172
	2	13.127	32.170	89.828	13.127	32.111	89.451
	5	13.021	31.910	89.109	13.021	31.854	88.743

**Table 4:** Effect of length scale parameter on dimensionless natural frequencies *Ω* based on the MSGT (nanoporosity-1, *m* = 1, *h* = 10 nm, *R* = 20*h*, *L*/*R* = 4, *e*_0_ = 0.5).

	*h*/*l* = 1	*h*/*l* = 1.5	*h*/*l* = 2	*h*/*l* = 3	*h*/*l* = 4	*h*/*l* = 5	*h*/*l* = 10
*n* = 1	0.25824	0.25723	0.25687	0.25662	0.25653	0.25649	0.25643
*n* = 2	0.20493	0.16730	0.15125	0.13840	0.13356	0.13125	0.12809
*n* = 3	0.42938	0.31512	0.25919	0.20833	0.18683	0.17591	0.16009
*n* = 4	0.76650	0.56684	0.46542	0.37079	0.32994	0.30894	0.27818
*n* = 5	1.17537	0.88269	0.72927	0.58336	0.51961	0.48665	0.43816
*n* = 6	1.64107	1.25206	1.04209	0.83851	0.74844	0.70162	0.63245

**Table 5:** Effect of the length scale parameter on dimensionless buckling load $\bar{F}$ based on the MSGT (nanoporosity-1, *m* = 1, *h* = 10 nm, *R* = 20*h*, *L*/*R* = 4, *e*_0_ = 0.5).

	*h*/*l* = 1	*h*/*l* = 1.5	*h*/*l* = 2	*h*/*l* = 3	*h*/*l* = 4	*h*/*l* = 5	*h*/*l* = 10
*n* = 1	0.12274	0.12167	0.12129	0.12101	0.12092	0.12087	0.12081
*n* = 2	0.04801	0.03188	0.02603	0.02177	0.02027	0.01957	0.01864
*n* = 3	0.18358	0.09853	0.06657	0.04297	0.03455	0.03062	0.02536
*n* = 4	0.55614	0.30326	0.20422	0.12951	0.10252	0.08987	0.07285
*n* = 5	1.27769	0.71906	0.49043	0.31364	0.24878	0.21820	0.17686
*n* = 6	2.45989	1.43011	0.99029	0.64101	0.51065	0.44875	0.36462

## Appendix A

The non-classical and classical resultant moments and forces in Equation (26) are:

(A1) $$N_{xx}=B_{11}\phi_{1}+A_{11}\phi_{0}+B_{12}\varphi_{1}+A_{12}\varphi_{0}$$

(A2) $$N_{yy}=B_{11}\varphi_{1}+A_{11}\varphi_{0}+B_{12}\phi_{1}+A_{12}\phi_{0}$$

(A3) $$N_{xy}=B_{55}k_{1}+A_{55}k_{0}$$

(A4) $$Q_{x}=K_{S}A_{55}\gamma_{1},Q_{y}=K_{S}A_{55}\gamma_{2}$$

(A5) $$M_{xx}=D_{11}\phi_{1}+B_{11}\phi_{0}+D_{12}\varphi_{1}+B_{12}\varphi_{0}$$

(A6) $$M_{yy}=D_{11}\varphi_{1}+B_{11}\varphi_{0}+D_{12}\phi_{1}+B_{12}\phi_{0}$$

(A7) $$M_{xy}=D_{55}k_{1}+B_{55}k_{0}$$

(A8) $$T_{xxx}=E_{1}\frac{\partial \phi_{0}}{\partial x}+E_{2}\frac{\partial \varphi_{0}}{\partial x}+\frac{E_{3}}{2}\frac{\partial k_{0}}{\partial y}+F_{1}\frac{\partial \phi_{1}}{\partial x}+F_{2}\frac{\partial \varphi_{1}}{\partial x}+\frac{F_{3}}{2}\frac{\partial k_{1}}{\partial y}$$

(A9) $$T_{yxx}=E_{2}\frac{\partial \varphi_{0}}{\partial y}+\frac{1}{2}\left(E_{4}\frac{\partial k_{0}}{\partial x}+F_{4}\frac{\partial k_{1}}{\partial x}\right)+E_{5}\frac{\partial \phi_{0}}{\partial y}+F_{2}\frac{\partial \varphi_{1}}{\partial y}+F_{5}\frac{\partial \phi_{1}}{\partial y}$$

(A10) $$T_{zxx}=E_{5}\phi_{1}+\frac{E_{4}}{2}\frac{\partial \gamma_{1}}{\partial x}+\frac{A_{1}}{2}\frac{\partial \gamma_{2}}{\partial y}+2A_{2}\varphi_{1}$$

(A11) $$T_{xyy}=\frac{E_{4}}{2}\frac{\partial k_{0}}{\partial y}+E_{2}\frac{\partial \phi_{0}}{\partial x}+E_{5}\frac{\partial \varphi_{0}}{\partial x}+\frac{F_{4}}{2}\frac{\partial k_{1}}{\partial y}+F_{2}\frac{\partial \phi_{1}}{\partial x}+F_{5}\frac{\partial \varphi_{1}}{\partial x}$$

(A12) $$T_{yyy}=E_{2}\frac{\partial \phi_{0}}{\partial y}+\frac{E_{3}}{2}\frac{\partial k_{0}}{\partial x}+E_{1}\frac{\partial \varphi_{0}}{\partial y}+F_{2}\frac{\partial \phi_{1}}{\partial y}+\frac{F_{3}}{2}\frac{\partial k_{1}}{\partial x}+F_{1}\frac{\partial \varphi_{1}}{\partial y}$$

(A13) $$T_{zyy}=\frac{A_{1}}{2}\frac{\partial \gamma_{1}}{\partial x}+2A_{2}\phi_{1}+E_{5}\varphi_{1}+\frac{E_{4}}{2}\frac{\partial \gamma_{2}}{\partial y}$$

(A14) $$T_{xxy}=\frac{E_{3}}{2}\frac{\partial \varphi_{0}}{\partial y}+\frac{E_{6}}{2}\frac{\partial k_{0}}{\partial x}+\frac{E_{4}}{2}\frac{\partial \phi_{0}}{\partial y}+\frac{F_{3}}{2}\frac{\partial \varphi_{1}}{\partial y}+\frac{F_{6}}{2}\frac{\partial k_{1}}{\partial x}+\frac{F_{4}}{2}\frac{\partial \phi_{1}}{\partial y}$$

(A15) $$T_{yyx}=\frac{E_{4}}{2}\frac{\partial \varphi_{0}}{\partial x}+\frac{E_{3}}{2}\frac{\partial \phi_{0}}{\partial x}+\frac{E_{6}}{2}\frac{\partial k_{0}}{\partial y}+\frac{F_{4}}{2}\frac{\partial \varphi_{1}}{\partial x}+\frac{F_{3}}{2}\frac{\partial \phi_{1}}{\partial x}+\frac{F_{6}}{2}\frac{\partial k_{1}}{\partial y}$$

(A16) $$T_{zxy}=A_{4}k_{1}+\frac{A_{5}}{2}\left(\frac{\partial \gamma_{2}}{\partial x}+\frac{\partial \gamma_{1}}{\partial y}\right)$$

(A17) $$T_{xxz}=\frac{A_{1}}{2}\varphi_{1}+\frac{A_{3}}{2}\frac{\partial \gamma_{2}}{\partial y}+\frac{E_{6}}{2}\frac{\partial \gamma_{1}}{\partial x}+\frac{E_{4}}{2}\phi_{1}$$

(A18) $$T_{yxz}=A_{4}\frac{\partial \gamma_{1}}{\partial y}+\frac{A_{5}}{2}\left(\frac{\partial \gamma_{2}}{\partial x}+k_{1}\right)$$

(A19) $$T_{xyz}=A_{4}\frac{\partial \gamma_{2}}{\partial x}+\frac{A_{5}}{2}\left(\frac{\partial \gamma_{1}}{\partial y}+k_{1}\right)$$

(A20) $$T_{yyz}=\frac{A_{1}}{2}\phi_{1}+\frac{A_{3}}{2}\frac{\partial \gamma_{1}}{\partial x}+\frac{E_{6}}{2}\frac{\partial \gamma_{2}}{\partial y}+\frac{E_{4}}{2}\varphi_{1}$$

(A21) $$M_{xxx}=F_{1}\frac{\partial \phi_{0}}{\partial x}+F_{2}\frac{\partial \varphi_{0}}{\partial x}+\frac{F_{3}}{2}\frac{\partial k_{0}}{\partial y}+G_{1}\frac{\partial \phi_{1}}{\partial x}+G_{2}\frac{\partial \varphi_{1}}{\partial x}+\frac{G_{3}}{2}\frac{\partial k_{1}}{\partial y}$$

(A22) $$M_{yxx}=F_{2}\frac{\partial \varphi_{0}}{\partial y}+F_{5}\frac{\partial \phi_{0}}{\partial y}+\frac{F_{4}}{2}\frac{\partial k_{0}}{\partial x}+G_{2}\frac{\partial \varphi_{1}}{\partial y}+G_{5}\frac{\partial \phi_{1}}{\partial y}+\frac{G_{4}}{2}\frac{\partial k_{1}}{\partial x}$$

(A23) $$M_{xyy}=\frac{F_{4}}{2}\frac{\partial k_{0}}{\partial y}+F_{2}\frac{\partial \phi_{0}}{\partial x}+F_{5}\frac{\partial \varphi_{0}}{\partial x}+\frac{G_{4}}{2}\frac{\partial k_{1}}{\partial y}+G_{2}\frac{\partial \phi_{1}}{\partial x}+G_{5}\frac{\partial \varphi_{1}}{\partial x}$$

(A24) $$M_{yyy}=F_{2}\frac{\partial \phi_{0}}{\partial y}+\frac{F_{3}}{2}\frac{\partial k_{0}}{\partial x}+F_{1}\frac{\partial \varphi_{0}}{\partial y}+G_{2}\frac{\partial \phi_{1}}{\partial y}+\frac{G_{3}}{2}\frac{\partial k_{1}}{\partial x}+G_{1}\frac{\partial \varphi_{1}}{\partial y}$$

(A25) $$M_{xxy}=\frac{F_{3}}{2}\frac{\partial \varphi_{0}}{\partial y}+\frac{F_{6}}{2}\frac{\partial k_{0}}{\partial x}+\frac{F_{4}}{2}\frac{\partial \phi_{0}}{\partial y}+\frac{G_{3}}{2}\frac{\partial \varphi_{1}}{\partial y}+\frac{G_{6}}{2}\frac{\partial k_{1}}{\partial x}+\frac{G_{4}}{2}\frac{\partial \phi_{1}}{\partial y}$$

(A26) $$M_{yyx}=\frac{F_{4}}{2}\frac{\partial \varphi_{0}}{\partial x}+\frac{F_{3}}{2}\frac{\partial \phi_{0}}{\partial x}+\frac{F_{6}}{2}\frac{\partial k_{0}}{\partial y}+\frac{G_{4}}{2}\frac{\partial \varphi_{1}}{\partial x}+\frac{G_{3}}{2}\frac{\partial \phi_{1}}{\partial x}+\frac{G_{6}}{2}\frac{\partial k_{1}}{\partial y}$$

## Appendix B

The parameters in Equations (42)–(46) are given by:

(A27) $$A_{11}=\int_{-\frac{h}{2}}^{\frac{h}{2}}(\lambda +2\mu )dz,B_{11}=\int_{-\frac{h}{2}}^{\frac{h}{2}}(\lambda +2\mu )zdz,\;D_{11}=\int_{-\frac{h}{2}}^{\frac{h}{2}}(\lambda +2\mu )z^{2}dz$$

(A28) $$A_{12}=\int_{-\frac{h}{2}}^{\frac{h}{2}}\lambda dz,B_{12}=\int_{-\frac{h}{2}}^{\frac{h}{2}}\lambda zdz,D_{12}=\int_{-\frac{h}{2}}^{\frac{h}{2}}\lambda z^{2}dz$$

(A29) $$A_{55}=\int_{-\frac{h}{2}}^{\frac{h}{2}}\mu dz,\;B_{55}=\int_{-\frac{h}{2}}^{\frac{h}{2}}\mu zdz,D_{55}=\int_{-\frac{h}{2}}^{\frac{h}{2}}\mu z^{2}dz$$

(A30) $$A_{1}=\int_{-\frac{h}{2}}^{\frac{h}{2}}a_{1}\;dz,\;A_{2}=\int_{-\frac{h}{2}}^{\frac{h}{2}}a_{2}\;dz\;,\;A_{3}=\int_{-\frac{h}{2}}^{\frac{h}{2}}a_{3}\;dz$$

(A31) $$A_{4}=\int_{-\frac{h}{2}}^{\frac{h}{2}}a_{4}dz,A_{5}=\int_{-\frac{h}{2}}^{\frac{h}{2}}a_{5}dz,B_{1}=\int_{-\frac{h}{2}}^{\frac{h}{2}}a_{1}zdz$$

(A32) $$\;B_{2}=\int_{-\frac{h}{2}}^{\frac{h}{2}}a_{2}zdz,\;B_{3}=\int_{-\frac{h}{2}}^{\frac{h}{2}}a_{3}zdz,\;B_{4}=\int_{-\frac{h}{2}}^{\frac{h}{2}}a_{4}zdz$$

(A33) $$\;E_{1}=\int_{-\frac{h}{2}}^{\frac{h}{2}}\beta_{1}dz,E_{2}=\int_{-\frac{h}{2}}^{\frac{h}{2}}\beta_{2}dz,\;E_{3}=\int_{-\frac{h}{2}}^{\frac{h}{2}}\beta_{3}dz$$

(A34) $$E_{4}=\int_{-\frac{h}{2}}^{\frac{h}{2}}\beta_{4}dz,E_{5}=\int_{-\frac{h}{2}}^{\frac{h}{2}}\beta_{5}dz,E_{6}=\int_{-\frac{h}{2}}^{\frac{h}{2}}\beta_{6}dz$$

(A35) $$F_{1}=\int_{-\frac{h}{2}}^{\frac{h}{2}}\beta_{1}zdz,F_{2}=\int_{-\frac{h}{2}}^{\frac{h}{2}}\beta_{2}zdz,\;F_{3}=\int_{-\frac{h}{2}}^{\frac{h}{2}}\beta_{3}zdz$$

(A36) $$F_{4}=\int_{-\frac{h}{2}}^{\frac{h}{2}}\beta_{4}zdz,F_{5}=\int_{-\frac{h}{2}}^{\frac{h}{2}}\beta_{5}zdz,\;F_{6}=\int_{-\frac{h}{2}}^{\frac{h}{2}}\beta_{6}zdz$$

(A37) $$G_{1}=\int_{-\frac{h}{2}}^{\frac{h}{2}}\beta_{1}z^{2}dz,G_{2}=\int_{-\frac{h}{2}}^{\frac{h}{2}}\beta_{2}z^{2}dz,G_{3}=\int_{-\frac{h}{2}}^{\frac{h}{2}}\beta_{3}z^{2}dz$$

(A38) $$G_{4}=\int_{-\frac{h}{2}}^{\frac{h}{2}}\beta_{4}z^{2}dz,G_{5}=\int_{-\frac{h}{2}}^{\frac{h}{2}}\beta_{5}z^{2}dz,G_{6}=\int_{-\frac{h}{2}}^{\frac{h}{2}}\beta_{6}z^{2}dz$$

## Appendix C

The nonzero components in Equations (53)–(55) are:

(A39) $$K_{11}=A_{11}\alpha_{m}^{2}+E_{1}\alpha_{m}^{4}+\left[A_{55}+\left(E_{3}+E_{4}+E_{5}+E_{6}\right)\alpha_{m}^{2}\right]\frac{n^{2}}{R^{2}}+\frac{E_{6}n^{4}}{2R^{4}}$$

(A40) $$K_{12}=K_{21}=\frac{\alpha_{m}n}{R}\left[A_{12}+A_{55}+\left(\frac{2E_{2}+E_{3}+E_{4}+E_{6}}{2}\right)\left(\alpha_{m}^{2}+\frac{n^{2}}{R^{2}}\right)\right]$$

(A41) $$K_{13}=K_{31}=-\frac{\left(E_{3}+E_{4}\right)\alpha_{m}n^{2}}{2R^{3}}-\frac{E_{2}\alpha_{m}\left(\alpha_{m}^{2}+\frac{n^{2}}{R^{2}}\right)}{R}-\frac{A_{12}\alpha_{m}}{R}$$

(A42) $$K_{14}=K_{41}=F_{1}\alpha_{m}^{4}+\frac{F_{6}}{2}\frac{n^{4}}{R^{4}}+\left(\frac{2F_{3}+2F_{4}+2F_{5}+F_{6}}{2}\right)\frac{\alpha_{m}^{2}n^{2}}{R^{2}}+B_{11}\alpha_{m}^{2}+\frac{B_{55}n^{2}}{R^{2}}$$

(A43) $$K_{15}=K_{51}=\left(\frac{2F_{2}+F_{3}+F_{4}+F_{6}}{2}\right)\left(\frac{\alpha_{m}^{3}n}{R}+\frac{\alpha_{m}n^{3}}{R^{3}}\right)+\left(B_{12}+B_{55}\right)\frac{\alpha_{m}n}{R}$$

(A44) $$\begin{array}{ll} K_{22}= & \frac{E_{6}\alpha_{m}^{4}}{2}+\left(\frac{E_{6}+2E_{3}+2E_{4}+2E_{5}}{2}\right)\frac{\alpha_{m}^{2}n^{2}}{R^{2}}+\frac{E_{1}n^{4}}{R^{4}}\\ & +\left(A_{11}+\frac{E_{6}}{R^{2}}\right)\frac{n^{2}}{R^{2}}+\alpha_{m}^{2}\left(A_{55}+\frac{A_{4}}{R^{2}}\right)+\frac{K_{S}A_{55}}{R^{2}} \end{array}$$

(A45) $$\begin{array}{ll} K_{23}=K_{32}= & -\frac{\left(2A_{3}+2A_{4}+A_{5}+E_{3}+E_{4}+2E_{5}\right)\alpha_{m}^{2}n}{2R^{2}}\\ & -\frac{\left(E_{1}+E_{6}\right)n^{3}}{R^{4}}-\frac{n}{R^{2}}\left(A_{11}+K_{S}A_{55}\right) \end{array}$$

(A46) $$\begin{array}{ll} K_{24}=K_{42}= & -\frac{\alpha_{m}n}{R^{2}}\left(A_{1}+A_{3}+A_{5}\right)\\ & +\left[\left(\frac{2F_{2}+F_{3}+F_{4}+F_{6}}{2}\right)\left(\frac{\alpha_{m}^{3}n}{R}+\frac{\alpha_{m}n^{3}}{R^{3}}\right)+\left(B_{12}+B_{55}\right)\frac{\alpha_{m}n}{R}\right] \end{array}$$

(A47) $$\begin{array}{ll} K_{25} & =K_{52}=-\frac{1}{2R}\left[\left(2A_{4}+A_{5}\right)\alpha_{m}^{2}+\left(E_{4}+E_{6}\right)\frac{n^{2}}{R^{2}}\right]-\frac{K_{S}A_{55}}{R}\\ & +\left[\frac{F_{6}\alpha_{m}^{4}}{2}+\frac{F_{1}n^{4}}{R^{4}}+\left(\frac{2F_{3}+2F_{4}+2F_{5}+F_{6}}{2}\right)\frac{\alpha_{m}^{2}n^{2}}{R^{2}}\right]+B_{55}\alpha_{m}^{2}+\frac{B_{11}n^{2}}{R^{2}} \end{array}$$

(A48) $$\begin{array}{ll} K_{33}= & \left(A_{3}+2A_{4}+A_{5}\right)\frac{\alpha_{m}^{2}n^{2}}{R^{2}}+\frac{\left(2E_{1}+E_{6}\right)n^{2}}{2R^{4}}+\frac{\left(2E_{5}+A_{3}\right)\alpha_{m}^{2}}{2R^{2}}\\ & +K_{S}A_{55}\left(\alpha_{m}^{2}+\frac{n^{2}}{R^{2}}\right)\;+\frac{E_{6}}{2}\left(\alpha_{m}^{4}+\frac{n^{4}}{R^{4}}\right)+\frac{A_{11}}{R^{2}} \end{array}$$

(A49) $$\begin{array}{ll} K_{34}=K_{43}= & \frac{\alpha_{m}\left(A_{1}+A_{3}\right)}{2R^{2}}+\alpha_{m}\left(K_{S}A_{55}-\frac{2F_{2}+F_{3}+F_{4}}{2R}\frac{n^{2}}{R^{2}}+\frac{E_{6}+E_{4}}{2}\alpha_{m}^{2}\right)\\ & +\frac{1}{2}\left(A_{1}+A_{3}+2A_{4}+3A_{5}\right)\frac{\alpha_{m}n^{2}}{R^{2}}+\frac{\alpha_{m}^{3}F_{2}}{R}-\frac{B_{12}\alpha_{m}}{R} \end{array}$$

(A50) $$\begin{array}{ll} K_{35}= & K_{53}=\frac{1}{2}\left[\left(A_{1}+A_{3}+2A_{4}+3A_{5}\right)\alpha_{m}^{2}+\left(E_{4}+E_{6}\right)\frac{n^{2}}{R^{2}}\right]\frac{n}{R}\\ & -\frac{F_{3}-F_{4}-2F_{5}}{2R}\frac{\alpha_{m}^{2}n}{R}-\frac{F_{1}}{R}\frac{n^{3}}{R^{3}}\;-\left(\frac{B_{11}}{R}-K_{S}A_{55}\right)\frac{n}{R} \end{array}$$

(A51) $$\begin{array}{ll} K_{44}= & G_{1}\alpha_{m}^{4}+\left[\left(\frac{2G_{3}+2G_{4}+2G_{5}+G_{6}}{2}\right)\frac{n^{2}}{R^{2}}+D_{11}+\frac{2E_{4}+2E_{5}+E_{6}}{2}\right]\alpha_{m}^{2}\\ & +\frac{G_{6}n^{4}}{2R^{4}}+\frac{n^{2}}{R^{2}}\left(D_{55}+2A_{4}+A_{5}\right)+K_{S}A_{55} \end{array}$$

(A52) $$\begin{array}{ll} K_{45}=K_{54}= & \frac{\alpha_{m}}{R}\left[\frac{2G_{2}+G_{3}+G_{4}+G_{6}}{2}\left(\alpha_{m}^{2}+\frac{n^{2}}{R^{2}}\right)\right]\\ & +\frac{\alpha_{m}}{R}\left(D_{12}+D_{55}+\frac{2A_{1}+4A_{2}+A_{3}+2A_{4}+3A_{5}}{2}\right) \end{array}$$

(A53) $$\begin{array}{ll} K_{55}= & \frac{G_{1}n^{4}}{R^{4}}+\frac{\alpha_{m}^{4}G_{6}}{2}+\left(\frac{2G_{3}+2G_{4}+2G_{5}+G_{6}}{2}\right)\frac{\alpha_{m}^{2}n^{2}}{R^{2}}\\ & +\frac{n^{2}}{R^{2}}\left(D_{11}+\frac{2E_{4}+2E_{5}+E_{6}}{2}\right)\;+\alpha_{m}^{2}\left(D_{55}+2A_{4}+A_{5}\right)+K_{S}A_{55} \end{array}$$

(A54) $$K_{g_{33}}=-\frac{m^{2}\pi^{2}}{L^{2}}$$

(A55) $$M_{11}=M_{22}=M_{33}=I_{0},M_{44}=M_{55}=I_{2},M_{14}=M_{25}=M_{41}=M_{52}=I_{1}$$
